# Interaction Between Heparan Sulfate Oligosaccharide and the Receptor-Binding Domain of the Wild-Type and Omicron Variant of the SARS-CoV-2 Spike Protein

**DOI:** 10.3390/biom15091343

**Published:** 2025-09-19

**Authors:** Marco Mandalari, Michela Parafioriti, Minghong Ni, Francesca Benevelli, Monica Civera, Stefano Elli, Marco Guerrini

**Affiliations:** 1 Dipartimento di Chimica, Università degli Studi di Milano, Via Golgi 19, 20133 Milan, Italy; marco.mandalari@unimi.it; 2 Istituto di Ricerche Chimiche e Biochimiche ‘G. Ronzoni’, via Giuseppe Colombo 81, 20133 Milan, Italy; parafioriti@ronzoni.it (M.P.); niminghong@ronzoni.it (M.N.); guerrini@ronzoni.it (M.G.); 3 BioSpin Business Unit, Bruker Italia S.r.l., Viale Vincenzo Lancetti 43, 20158 Milan, Italy; francesca.benevelli@bruker.com

**Keywords:** SARS-CoV-2, recognition protein S spike, heparan sulfate, heparin, glycosylation, molecular recognition, ^1^H-STD NMR spectroscopy, molecular dynamic simulation

## Abstract

Heparan sulfate proteoglycans serve as initial attachment sites for several viruses and bacteria. Recent studies suggest that SARS-CoV-2 similarly exploits these glycosaminoglycans, facilitating conformational changes in the spike protein that promote the interaction between the receptor-binding domain (S1-RBD) and the cellular angiotensin-converting enzyme 2 receptor (ACE2), thereby triggering the virus internalization process. The molecular details that drive this process, particularly the co-receptor role of heparan sulfate (HS), remain incompletely understood. The interaction between an HS hexasaccharide (hexa) and the N343 glycosylated S1-RBD of the wild-type (WT) and Omicron variant of SARS-CoV-2 was investigated. The conformational properties of hexa with these S1-RBDs in unbound and bound states are explored using multiple independent MD simulations; the protein binding epitope of hexa, as well as the details of its interaction with S1-RBD of the Omicron variant, are characterized by comparing experimental and theoretical ^1^H STD NMR signals. This investigation identifies the role played by the glycosyl moiety at N343 in potentially affecting this interaction in both WT and Omicron S1-RBD, explaining the observed low specificity and multi-modal nature of the interaction between HS oligosaccharides and these S1-RBDs.

## 1. Introduction

Heparan sulfate (HS) and the structurally similar heparin are linear, sulfated polysaccharides belonging to the glycosaminoglycan (GAG) family. Their underlying repeating unit consists of a disaccharide composed of a uronic acid (β-D-glucuronic) 1→4 linked to α-D-glucosamine (GlcN). This backbone undergoes modification [1], including partial N-deacetylation/N-sulfation, epimerization (converting a proportion of β-D-glucuronic to α-L-iduronic residues), and O-sulfation, resulting in a heterogeneous population of linear chains with variable length and composition. Heparan sulfate and heparin vary in terms of the extent of N-acetylation/N-sulfation, their levels of 6-O-, and, less commonly, 3-O-sulfation of glucosamine units and 2-O-sulfation of uronic acid residues [2,3,4]. In contrast with HS, heparin is characterized by shorter chains, more extensive sulfation, and higher iduronic acid content.

Heparan sulfate is organized in proteoglycans (HSPGs) or glycoproteins, which are expressed ubiquitously on the cell surface and within the extracellular matrix [2]. The polyanionic nature of HS, arising from its carboxylate and sulfate groups, enables it to interact with a broad spectrum of proteins, including cytokines, chemokines [5], growth factors [6], microbial adhesion factors [7], viral and/or bacterial recognition proteins [8], which are characterized by clusters of solvent exposed positively charged Lys and/or Arg side chains. An analysis of the heparin binding proteins found that these clusters are most commonly formed from triple amino acids—at least one of Arg or Lys, but also a hydrogen bonding amino acid and a non-polar amino acid [9].

Recent attention has focused on the role of HS in viral pathogenesis, particularly in the context of emerging respiratory viruses. Several pathogens, including herpes simplex virus [10], Dengue virus [11], and respiratory syncytial virus [12,13,14], exploit HS to initiate their contact with host cells. Experimental evidence suggests that SARS-CoV-2 also utilizes HS as an auxiliary attachment factor [15]. This virus, characterized by its single-stranded RNA genome and a lipid capsid studded with spike (S) glycoproteins, employs its receptor-binding domain within the S1 subunit of the RBD (S1-RBD) to engage angiotensin-converting enzyme 2 (ACE2), which is located on the surface of the host cells. While ACE2 serves as the primary entry receptor, structural and biochemical studies have indicated that initial interactions between the spike protein and cellular HS facilitate viral engagement with ACE2 [16,17] through the formation of a ternary complex comprising S1-RBD, HS, and ACE2 [15,18].

The emergence of SARS-CoV-2 variants with enhanced transmissibility and altered receptor affinities requires an understanding of how SARS-CoV-2 spike (S) protein variants interact with HS. The Omicron variant harbors multiple mutations in the S1-RBD, most of which are proximal to the ACE2 attachment site, which may modulate its interaction with co-receptors like HS and ACE2 [19]. Significant differences, in terms of both kinetic and thermodynamic aspects of the interaction between heparin and wild-type (WT), Delta, and Omicron S1-RBDs, have been observed [20], but the structural causes and functional implications of these changes remain poorly defined.

A similar situation obtains for glycosylation, which is a crucial post-translational modification that shapes protein structure and function [21]. The S protein of SARS-CoV-2 is heavily glycosylated, forming a glycan shield that masks over 60% of its surface, helping the virus to evade detection by the immune system [22]. It has been shown that the N331 and N343 residues are two highly conserved glycosylation sites in the S1-RBD region present across all the variants of SARS-CoV-2 RBDs [23]. While N331 is located in the highly flexible region connecting the S1-RBD to the N-terminal domain of the protein S, the N343 glycan extends across the S1-RBD surface, bridging the two helical regions flanking the central beta-sheet core. It has been proposed that it may interfere with a significant portion of the domain that binds HS in the S1-RBD [22,24].

**Figure 1:**
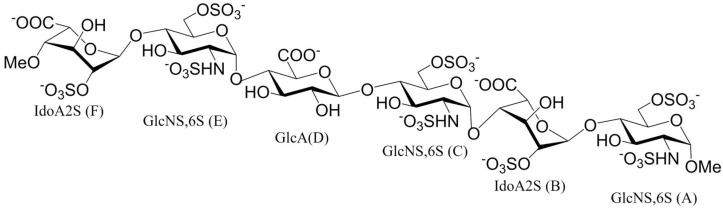
Structure of the hexasaccharide (hexa). The monosaccharide units are labeled by the name of the residue: IdoA2S, 2-O-sulfated iduronic acid; GlcNS,6S, N-sulfated, 6-O-sulfated glucosamine; GlcA, glucuronic acid. Capital letters denote residues from F (non-reducing end) to A (reducing end).

In this study, the molecular interaction between the HS hexasaccharide (hexa, Figure 1) and the S1-RBD of both the WT and Omicron SARS-CoV-2 variants was investigated. In parallel, the interference between the glycosyl moiety at position N343 and the HS binding site of S1-RBDs has been characterized. Multiple independent molecular dynamics (MD) simulations were applied to predict the glycan conformation in the unbound and bound states, as well as the geometry of the complexes formed between hexa and the Omicron (Omi) and WT S1-RBDs, which have been generated previously by docking [18]. Proton saturation transfer difference (^1^H-STD) NMR spectroscopy allows for the experimental validation of Omi-S1-RBD-hexa complexes and the corresponding protein epitope binding. These signals were acquired as *STD_0_*, which corresponds to the initial slope of the curve, and ^1^H-STD % vs. mixing time of the experiment, and for that reason, they represent an STD intensity less affected by the re-binding phenomena of the system. Furthermore, selected *STD_0_* were compared with the corresponding simulated values using the RedMat application [25]. In summary, this study characterizes the structural and dynamic aspects of the interaction between the HS oligosaccharide and Omicron and the WT S1-RBD, as well as exploring the potential role of the N343 glycosyl moiety in perturbing this contact. The findings enhance our understanding of the molecular mechanism by which SARS-CoV-2 takes advantage of cell surface HS to interact and initiate infection of the cell host, and they will assist the design of new antiviral strategies against SARS-CoV-2.

## 2. Materials and Methods

### 2.1. Molecular Dynamics Simulations

#### 2.1.1. System Preparation and MD Simulation Protocol

The initial geometries that were submitted to MD simulations were selected from the docking results reported in our previous work [26], where the S1-RBD sequence employed in the study covered amino acids 333–526; therefore, the glycosylation at the N331 site is not considered.

For the wild-type S1-RBD system (referred to as WT-S1-RBD-hexa), we started from the single complex structure that was obtained previously [26], while for the Omicron S1-RBD system, the two top-ranked poses, which represent the two principal binding modes, were selected (referred to as Omi-S1-RBD-hexa-I and Omi-S1-RBD-hexa-II, respectively) (Figure S1). Amber 18 [27] software was used for the systems preparation, MD simulations, and analysis. We applied the Amber (ff14SB) [28] and the GLYCAM-06j-1 [29] force fields for the protein and ligand, respectively. The glycosylated moiety at N343 was also described with the GLYCAM-06j-1 force field for carbohydrates. The hexa oligosaccharide was described in its fully deprotonated form, since at pH = 7.0, all the ionizable groups are dissociated.

Each system was solvated with a TIP3P [30] cubic water box edge of 15 Å. Na^+^ ions were added to neutralize the system, and then Na^+^Cl^−^ ions were added to mimic the ionic strength of a physiological environment ([NaCl] = 0.15 M). The cut-off for no-bond interaction was set to 8 Å; the Particle Mesh Ewald method was used to treat long-range electrostatic interactions. Each system was minimized using three consecutive minimization steps, each consisting of 20 cycles of the steepest descent algorithm followed by 880 cycles of conjugate gradient minimization. Harmonic restraints (k = 50 and k = 10 Kcal mol^−1^ × Å^−2^) were applied to solute atoms relative to the input geometry in these first two steps, while the final step was performed without restraints, allowing full minimization.

The systems were then heated to 300 K in 100 ps in NVT conditions (Langevin thermostat [31] with collision frequency $\gamma =1\;ps^{-1}$) with a time step of 0.5 fs. The hydrogen atoms were fixed using the SHAKE [32] algorithm, and a positional restraint of 10 Kcal mol^−1^ × Å^−2^ was set for the solute atoms. To equilibrate the system density, we first performed an equilibration step in NVT conditions (100 ps, Δt = 0.5 fs) with restrained solute atoms (5 Kcal mol^−1^ × Å^−2^), followed by an equilibration run in NPT conditions (Berendsen Barostat [33], *p* = 1 atm, Δt = 0.5 fs, and pressure relaxation time $\tau_{P}$ = 2 ps) and a final NVT step with no restraints (100 ps, Δt = 0.5 fs). After equilibration at T = 300 K, MD simulations were carried out in triplicate. The production runs (200 ns, Δt = 2 fs) were performed in NVT conditions using the Langevin thermostat [31] to maintain a stable temperature of 300 K. All the replicas had the same coordinates but different velocities, randomly selected from a Maxwell distribution at 300 K. Frame coordinates were saved every 20 ps for a total of 10,000 structures for each run. For the analysis, each of the three independent replicas was merged into one single meta-trajectory, including 60,000 sampled geometries.

The oligosaccharide hexa in the unbound state was characterised by three independent MD simulations, following the same protocol conditions previously described for the hexa in the bound state with the S1-RBD. For one of the replicas, the initial conformation of hexa was characterized by residues IdoA2S, GlcA, and GlcNS6S in chairs ^1^C_4_, ^4^C_1_, and ^4^C_1_, in that order. The initial glycosidic conformation was set as follows, $\phi_{1}/\psi_{1}$ = 41°/14°, $\phi_{2}/\psi_{2}$ = −40°/−30°, $\phi_{3}/\psi_{3}$ = 60°/30°, $\phi_{4}/\psi_{4}$ = −39°/−33°, and $\phi_{5}/\psi_{5}$ = 41°/14°, in order from the non-reducing to reducing end, as reported in our previous work [18]. The glycosidic dihedral angles are defined as $\phi_{i}={H1}_{n}\to {C1}_{n}\to {O4}_{n}\to {C4}_{n+1}$ and $\psi_{i}={C1}_{n}\to {O4}_{n}\to {C4}_{n+1}\to {H4}_{n+1}$. The hexa initial geometries for the second and third MD simulation replicas were obtained by adding or subtracting 10°, respectively, to the initial $\phi_{i}$ and $\psi_{i}$ values defined previously. The MD simulation trajectories of these three independent replicas were merged into one meta-trajectory that includes 60,000 sampled geometries of hexa for the analysis. Each meta-trajectory (hexa in unbound and bound states with S1-RBD) was analyzed using CPPTRAJ [34].

#### 2.1.2. Filtering and Analysis of the MD Trajectories: Bound State Condition of S1-RBD and Hexa

We filtered the meta trajectories of each hexa-S1-RBD complex to select the frames in which the oligosaccharide hexa is in the bound state with the corresponding S1-RBD. Precisely, the hexa is in the bound state with S1-RBD, if and only if the center of mass (CoM) of the two central monosaccharides (C) and (D) is within 8 Å from the CoM of the proximal amino acid (MDAnalysis 8 Library tool v 2.8.0 Python v 3.10.16). The percentage of frames of each meta-trajectory that agrees with this bound state condition was 73% for Omi-S1-RBD-hexa-I, 53% for Omi-S1-RBD-hexa-II, and 39% for WT-S1-RBD-hexa. The filtered MD simulation trajectories were analysed using CPPTRAJ [34].

#### 2.1.3. Model Validation by Reduced Matrix (RedMat)

The application RedMat 1.0 compares the experimental *STD_0_* measured upon interaction between hexa and Omicron S1-RBD with the corresponding theoretical values, which were simulated using selected geometries of the complexes between Omicron S1-RBD and hexa [25]. The following parameters were set for the simulation of *STD_0_*: a spectrometer frequency at 600 MHz, a correlation time ($\tau_{c})$ of the complex at 25 ns, and a cut-off distance of 15 Å. The analysis was performed on the meta-trajectory that was previously filtered using the bound state condition (see Section 2.1.2). To select representative ligand-binding modes, we carried out a ligand cluster analysis on those frames of the complexes Omi-S1-RBD-hexa-I and Omi-S1-RBD-hexa-II that exhibits an R-NOE factor below 0.3 after fitting on protein residues of the frames.

#### 2.1.4. Cluster Analysis

The cluster analysis was applied to the meta-trajectories upon filtering with the bound state condition and an R-NOE smaller than 0.3 in the complexes Omi-S1-RBD-hexa-I and Omi-S1-RBD-hexa-II. Differently, the cluster analysis was applied on the meta-trajectory upon filtering with only the bound state condition in complex WT-S1-RBD-hexa; in fact, no *STD_0_* were available in this last case.

The cluster analysis was performed using CPPTRAJ v.18.01 [34], considering all carbon, oxygen, sulfur, and nitrogen atoms for the hexa ligand. Clusters are generated using the hierarchical agglomerative approach, setting a cut-off distance of 10 Å between the clusters. The distance is calculated as the average distance between members of two clusters. A cluster is considered representative if the population percentage is not less than 10%.

### 2.2. NMR Spectroscopy: Sample Preparation and Experimental Procedure

The S1-RBD of the Omicron variant, expressed in HEK293 cells, was purchased from GenScript (Rijswijk, The Netherlands). The protein solution (1.12 mg/mL in phosphate buffer pH 7.4) was washed with 20 mM HEPES-d18 pH 7.2 with 200 mM NaCl (D_2_O) using VWR® centrifugal filters (10 kDa membrane, 0.5 mL).

The NMR experiments were performed with a Bruker Avance III spectrometer (Bruker BioSpin AG, Fällanden, Switzerland) operating at 600.13 MHz and equipped with a high-sensitivity 5 mm TCI cryoprobe. The ligand was dissolved in 0.2 mL of the purified protein, and the final solution was transferred into a 3 mm NMR tube. The concentrations of ligand and protein were 5.4 μM and 540 μM, respectively (protein/ligand ratio = 1:100). The spectra were acquired at 293 K. Based on our previous work [18], the number of scans was 2048, the dummy scans were 4, and the relaxation delay was set to 6 s. A spin-lock pulse of 10 ms was employed to remove the protein broad signal. The selected frequencies for acquiring the on- and off-resonance spectra were 580 and 20,000 Hz, respectively. Five different experiments were performed, varying the saturation time (0.5, 1, 2, 3, 5 s). The ^1^H-STD NMR spectra were obtained by phase cycling subtraction of the on-resonance and off-resonance data acquired in interleaved mode. The STD intensities at the initial slope were calculated for the anomeric proton signals since they are sharp and well-resolved. A mono-exponential equation, as proposed by Mayer and James [35] (Equation (1)), was used to fit the experimental build-up curves.

(1) $$STD\left(t_{sat}\right)=STD^{max}\left(1-e^{-k_{sat}\cdot t_{sat}}\right)$$

The resulting values were expressed as relative *STD_0_* percentages by normalizing the intensities against the most intense signal.

## 3. Results and Discussion

The hexasaccharide (hexa) shown in Figure 1 was used as a molecular probe to characterize the interaction between HS and the tip of the wild-type (WT, PDB ID: 6M0J [36]) and the Omicron (PDB ID: 7WBP [37]) S1-RBDs of SARS-CoV-2 (Figure S1). In fact, the sequence of hexa represents the average composition of HS [2,4] chains and corresponds to the longer uninterrupted sequence of heparin residues binding WT S1-RBD [18] and its Omicron [26] variant, as detected by the ^1^H-STD experiment.

In our previous work [26], we observed that the S1-RBD of WT, Delta, and Omicron variants are characterized by different affinities for immobilized heparin. This suggests the hypothesis that mutations in the S1-RBD can modulate the association and dissociation process with HS, thereby affecting their interaction with the ACE2 receptor. Omicron S1-RBD has a stronger affinity for HS compared to the wild-type strain [20]. To characterize the interaction between hexa and Omicron S1-RBD, we applied a computational workflow combining MD simulations with ^1^H-STD NMR interaction experiments. Analogously, the interaction between hexa and WT S1-RBD was analyzed using MD simulation, since no experimental *STD_0_* were available for that system. Starting from the relevant binding modes generated previously by docking [26], we carried out multiple independent molecular dynamics (MD) simulations. Additionally, a full conformational analysis of hexa in unbound and in bound states with S1-RBDs of both WT and Omicron variant was conducted to detect potential conformational changes induced upon interaction with these S1-RBD variants.

### 3.1. ^1^H-STD NMR Interaction Experiment Between Hexa and Omi S1-RBD

**Figure 2:**
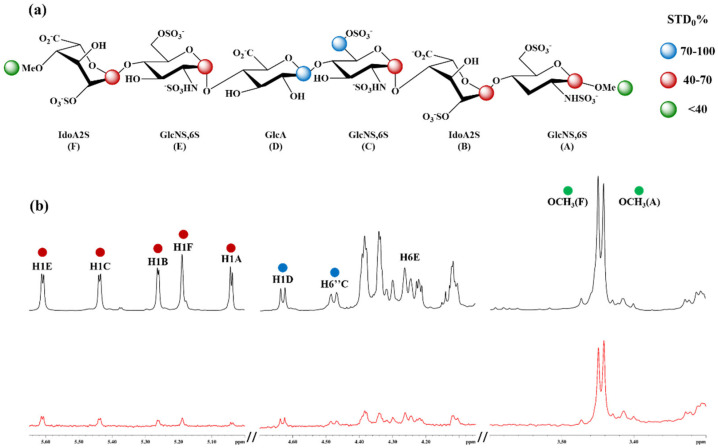
^1^H-STD NMR spectrum of the interaction between hexa and Omicron S1-RBD. Ligand epitope binding map of hexa in interaction with Omicron S1-RBD (**a**). Off-resonance (black line) and on-resonance (red line) spectra of the complex at saturation time equal to 3 s (T = 293K), with the assignment of the protons shown in (**b**).

^1^H-STD NMR experiments were conducted to identify the protons and residues of hexa that interact with Omicron S1-RBD, assessing their proximity to its surface. *STD_0_* percentages (*STD_0_* %) for non-overlapping ligand protons (i.e., anomeric protons) were calculated as described in the Materials and Methods section and subsequently mapped onto the ligand structure to depict the interacting epitope map (Figure 2 and Figure S2a). Comparison of the STD NMR spectra with the corresponding ^1^H spectra revealed that all anomeric protons of hexa exhibit significant STD effects, indicating that each sugar unit is in contact with the surface of S1-RBD (Figure 2). The highest *STD_0_* % effect was observed for H6C (100%), followed by H1D (96%), while H1F, H1E, H1C, H1B, and H1A displayed lower STD enhancements, ranging from 10% to 36% (Table S1). H6E also shows an appreciable contribution to the interaction (Figure 2 and Figure S2b). The corresponding *STD_0_*, however, was not determined owing to spectral overlap (Figure S2b).

It is notable that H6C and H6E present approximately opposite orientations in the 3D structure of hexa (Figure S3). The detection of these two signals at comparable intensities (Figure 2 and Figure S2b) indicates that hexa presumably interacts with site I of the S1-RBD (Figure S4) with opposite orientations of the glycan relative to the S1-RBD, corresponding to two different epitope binding modes, in which H6C or, alternatively, H6E faces the surface of S1-RBD [18].

### 3.2. Conformation of Hexa in the Bound State with Omicron and WT S1-RBD

**Figure 3:**
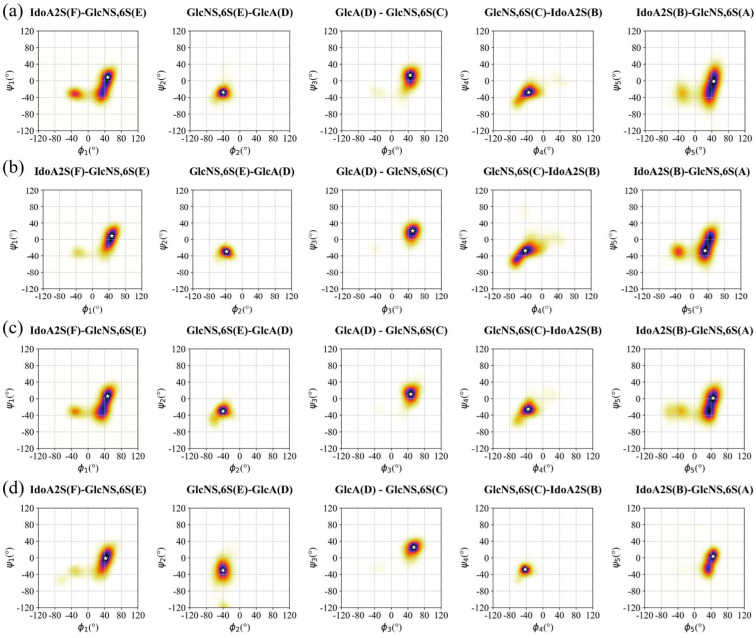
Ramachandran plot and density color maps of the hexa glycosidic angles $\phi_{i}/\psi_{i}$ in unbound and bound states with S1-RBDs. Hexa in unbound state (**a**); Omi S1-RBD-hexa-I (**b**); Omi S1-RBD-hexa-II (**c**); WT S1-RBD-hexa (**d**). The color gradient (yellow to blue) is proportional to the $\phi_{i}/\psi_{i}$ sampled state, identifying (qualitatively) the preferred conformations. The most populated $\phi_{i}/\psi_{i}$ state is represented as a white star.

The binding epitope of hexa with Omicron S1-RBD is characterized by strong ^1^H-STD signals originating from units C and D of the oligosaccharide in the bound state with the Omi-S1-RBD (Figure 2 and Table S1). This supports the idea that GlcNS6S(C) and GlcA(D) are, on average, closer to the protein surface in comparison to the terminal residues. Hexa, when bound to the Omi-S1-RBD or WT-S1-RBD, almost preserves the conformational properties that were observed in its unbound state. Specifically, residues A, C, E, and D adopt a ^4^C_1_ chair conformation, while IdoA2S (residues B and F) assume the ^1^C_4_, chair, even though a minor population of the skew-boat ^2^S_0_ is present (Figure S5 and Table S2). The inter-glycosidic bond geometry of hexa remains essentially unaltered upon interaction with the Omicron and WT S1-RBDs, preserving their unbound state conformation. This can be seen by comparing the most populated $\phi_{i}/\psi_{i}$ states in Figure 3a with Figure 3b–d (see also Figure S6 and Table S3).

This result is consistent with the structural and conformational properties of hexa, in which the strong electrostatic repulsion between sulfated and carboxylate groups, along with the corresponding steric hindrance, is dominant and forces the molecule to preserve its linear, relatively stiff conformation, which is typical of its unbound state, despite the interaction with different S1-RBDs. As a result, the inter-glycosidic bond geometries remain largely unaltered upon interaction with the surface of S1-RBD, suggesting weak and low specificity interaction between this oligosaccharide and these S1-RBDs. Interestingly, the structural rigidity of hexa preserves the position of its charged groups (sulfates and carboxylates) that remain oriented in approximately opposite directions. In summary, the conformation of hexa defines and preserves two wider faces, both potentially available to contact the positively charged patches that are solvent exposed in the site I of S1-RBDs (Figure S3).

### 3.3. Characterization of the Interaction Between Hexa and S1-RBD of Wild-Type and Omicron Variant

#### Analysis of the MD Simulation Meta-Trajectories

Starting from the most representative docking poses obtained in our previous study [26] (Figure S1), we ran three independent MD simulations for each of the modeled complexes, labeled as Omi-S1-RBD-hexa-I, Omi-S1-RBD-hexa-II, and WT-S1-RBD-hexa. In all of these systems, hexa remains preferentially bound to the shallow channel defined by residues R346, N354, R355, K356, R357, and R466, which were previously identified as site I [18,38] (Figure S4). Interestingly, the docking does not provide solutions in which hexa binds Omi-S1-RBD or WT-S1-RBD through site II (K424, R454, R457, K458, K462, and R466) and III (R403, R408, K417, and K444). According to the docking results, the Omicron S1-RBD complexes show hexa bound within sites I in two reverse orientations, allowing the two opposite surfaces of hexa (Figure S3) to interact with the surface of the S1-RBD. These two alternative modes of interaction are identified by the GlcNS6S(A) oriented toward the ACE2 binding site and proximal to R346 (Figure S1a). In contrast, when the opposite face of hexa approaches the site I of S1-RBD, residue A is in contact with R355 and R466 (Figure S1b). In the WT-S1-RBD-hexa, the oligosaccharide is docked in a single orientation in which residue A is proximal to R346 and K356, as shown in Omi-RBD-hexa-II (Figure S1c).

Analyzing the MD simulation meta-trajectories of the hexa in the bound state with Omicron and WT S1-RBD, the ligand exhibits significant mobility along the channel, corresponding to site I (Figure S7a–c), as detected by the RMSD, while the secondary structure of protein S1-RBD remains stable in the spanned simulation time (Figures S7d–f and S8). In fact, in all simulated hexa S1-RBD complexes, the RMSD (calculated on heavy atoms of hexa relative to the input structure) shows a significant change, ranging between 10 and 30 Å (Figure S7a–c). Since the ligand conformation remains stable throughout the simulations (Figure 3), this variation in RMSD values (Figure S7a–c) reflects only the rotational and translational movements that hexa experiences when in contact with the concave and shallow surface of site I of both Omicron and WT S1-RBD (Figure S9). Despite the multiple electrostatic interactions between the positively charged amino acids and the negative charges of the oligosaccharide, the ligand retains enough kinetic energy to explore a limited region surrounding site I on the surface of S1-RBD.

**Figure 4:**
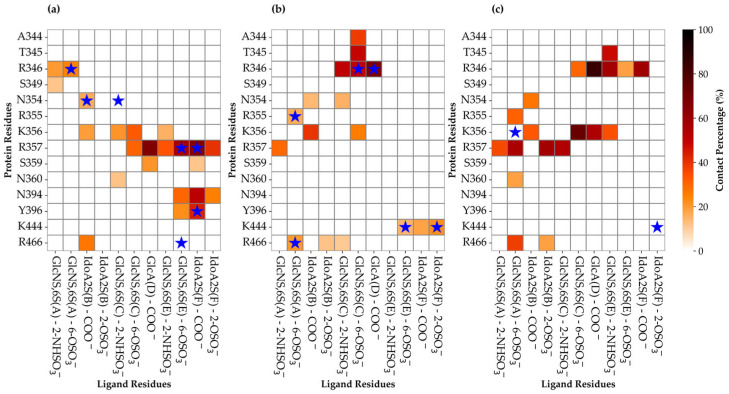
Heatmap plots of ligand-protein contacts. Heatmap plots of ligand–protein contacts established in Omi-S1-RBD-hexa-I (**a**), Omi-S1-RBD-hexa-II (**b**), and WT-S1-RBD-hexa (**c**). The color scale represents the frequency of each contact; dark red/black indicates greater persistence. The interactions observed in the docking pose (i.e., at the beginning of the MD simulation) are highlighted with a blue star.

To identify the amino acids that are in persistent contact with the ligand, we applied a 6 Å cut-off filter between the hexa heavy atoms and key residues of S1-RBD. The frequency of each contact was then calculated as a percentage of the whole meta-trajectory and reported in Figure 4 as contact heatmaps.

The color gradient in Figure 4 ranges from lighter shades (orange to white), indicating less frequent contacts, to darker shades (dark red or black), representing more persistent (stronger) interactions. If an atom of the oligosaccharide forms more than one contact with the same amino acid, then only the contact with the highest percentage was reported. For each complex, the blue stars indicate a contact that was already present in the initial pose. Furthermore, these interactions have been classified as ionic or polar, according to the atoms or groups that are involved. Contacts between positively and negatively charged groups are classified as electrostatic interactions. Contact involving polar atoms with an average distance ≤ 4.5 Å are predicted to be polar interactions and/or potential hydrogen bonds (Table S4).

The Omi-S1-RBD-hexa-I system exhibits alteration of its initial binding epitope, indicating that the bound glycan explores the surroundings of site I. The amino acids involved predominantly in this contact are R357 (67%), followed by Y396 (43%), K356 (32%), R466 (27%), N394 (25%), and R346 (20%). R357 makes the most frequent contacts with the hexa and, compared to the initial state, forms additional electrostatic interactions with glucosamine C, glucuronic acid D, and iduronic acid F, with frequencies ranging from 66% to 33% (Table S4). K356 forms new electrostatic interactions with the central part of the oligosaccharide (residue C). Two residues, S359 and N360, that are proximal to site I (Figure S4) and known to be involved in GAG-SARS-CoV-2 recognition [39], engage the central C and D residues of the glycan. Furthermore, a new polar contact involving N394 and Y396 and glycan residues E and F, respectively, was established, while the interaction between E and R466 was lost.

In the Omi-S1-RBD-hexa-II system, the contacts that were established upon docking are preserved, despite new interactions also being gained (Figure 4b). R346 persistently engages the central sugars C and D, showing contact percentages of 51% and 67%, respectively (Table S4c). These contacts are further stabilized by less populated polar interactions T345-GlcNS,6S(C) (49%) and GlcNS,6S(C)-A344 (38%). Furthermore, residues K356 and R357, which are part of the core of site I, frequently interact with IdoA2S (B) (40%) and GlcNS6S(A) (27%). Interestingly, the heatmaps in Figure 4a,b predict a different binding epitope for the hexa in the bound state with Omicron S1-RBD, through its two opposite surfaces (Figure S3), suggesting that these two surfaces present a comparable capacity to interact with the tested S1-RBDs.

In the WT-RBD system, the hexa moves across the central area of site I, displaying greater mobility than that observed in Omicron S1-RBD and hexa. This increased mobility is evident in the persistent contacts in Figure 4c, where they differ from the initial binding epitope, indicated by blue stars. R346 preserves contact with GlcA(D) (85%), IdoA2S(F) (60%), and GlcNS6S(E) (57%). Analogously, K356 and R357 form persistent electrostatic interactions with the central residue GlcNS6S(C), whose populations are 72% and 55%, respectively. Interestingly, K356 shows weaker contacts with terminal residues GlcNS,6S(E) and IdoA2S(B), while R357 shows contacts with IdoA2S(B) and GlcNS6S(A) (Table S4e–f). This reveals how the bound glycan spans the surface of the S1-RBD that surrounds site I in the simulated time window. Furthermore, N354, R355, K356, R357, N360, and R466 exhibit a range of contact frequencies between 57% and 18% with the terminal units IdoA2S(B) and GlcNS6S(A). Interestingly, the contacts between R346 and GlcA(D) or GlcNS6S(C); between K356 and IdoA2S(B) or GlcNS6S(C); between R355 and GlcNS6S(A), and between R357 and GlcNS6S(A) were previously found in Omi-S1-RBD-hexa-II.

An important finding that emerges from the contact analysis and the visual inspection of the MD simulation trajectories is the mobility of hexa when in the bound state with the Omicron S1-RBD and particularly with WT S1-RBD. In all of these systems, the mobility of hexa undergoes repeated engagement and disengagement of ion–pair interactions between the anionic functional group of the oligosaccharide and the cationic side chains of Arg and Lys that belong to site I and its surroundings. Specifically, the sulfo groups of glucosamines A, C, and E, as well as the carboxyl groups of iduronic acid B and F, establish electrostatic interactions with the same key positive and/or polar side chain of R346, K356, R357, and N394 in Omi-RBD-hexa-I (Figure 4a). This suggests that hexa alternately contacts these residues, moving through the shallow cavity of site I in the Omi-RBD-hexa-I system. Analogously, the same glycan that engages the surface of Omicron S1-RBD, but with opposite orientation, allows its sulfo and carboxyl groups to contact R355 and K356 in the Omi-RBD-hexa-II (Figure 4b) or K356 and R357 in the WT-RBD-hexa complex (Figure 4c). These results indicate noteworthy ligand mobility; the hexa undergoes a combination of rotation and translation movements on the positively charged surface, depicting a protein binding epitope that appears more extended than the size of the hexasaccharide probe.

### 3.4. The Mutation G339D in Omi-S1-RBD Reduces the Shielding Effect That the N343 Glycosyl Moiety Exerts on Site I

**Figure 5:**
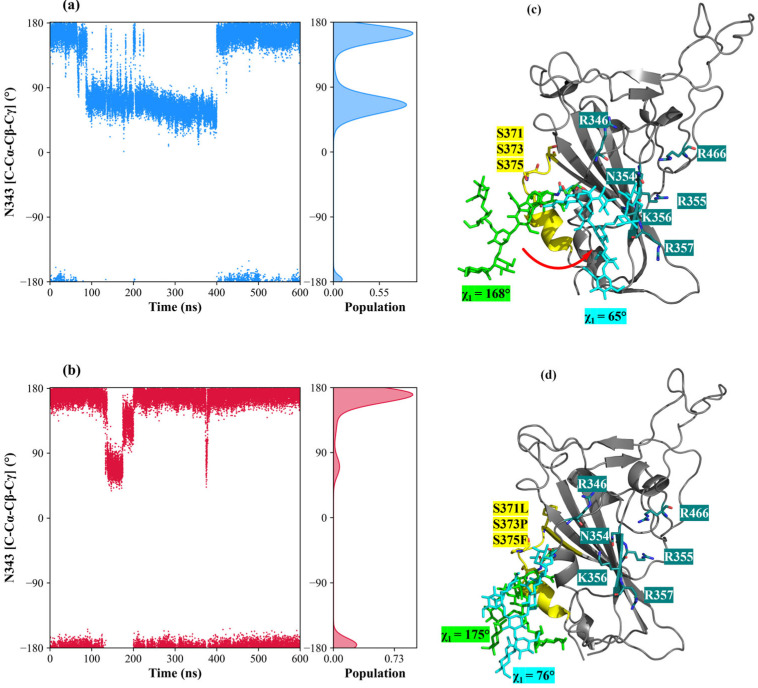
Conformational analysis of FA2G2 WT-S1-RBD-hexa (blue) and Omi-S1-RBD-hexa-I (red). (**a**,**b**) Scatter plot of the χ_1_ torsion angle of the N343 side chain in WT-S1-RBD-hexa (blue) and Omi-S1-RBD-hexa-I (red), with a histogram distribution plot along the y-axis. (**c**,**d**) Representative snapshots of WT-S1-RBD and Omi-S1-RBD-hexa-I, respectively. The protein is shown as a grey cartoon, helix 366–371 and loop 372–375 are highlighted in yellow (residues 371, 373, and 375 are displayed as sticks), and binding site residues R346, N354, R355, K356, R357, and R466 are highlighted in teal. The FA2G2 octasaccharide is represented as sticks: green indicates the population with χ_1_ ≈ 160°, while cyan corresponds to the population with χ_1_ ≈ 70°. In panel c, the conformational transition of FA2G2 from anti to gauche^+^ state is reported with a red arrow.

To elucidate the role that the N343 glycosyl moiety exerts on the interaction between hexa and S1-RBD, the corresponding oligosaccharide was built (by molecular editing) and attached to the Omicron and WT S1-RBDs [26]. This N-glycan corresponds to the biantennary core-fucosylated octasaccharide FA2G2 with the structure [GlcNAc^1^ (α1→6) Fuc^8^] (β1→4) GlcNAc^2^ (β1→4) Man^3^ [(β1→3) Man^4^ (α1→6) GlcNAc^5^] [(β1→6) Man^6^(α1→6) GlcNAc^7^], which has been identified as the most abundant glycan sequence across all SARS-CoV-2 spike proteins [40]. Interestingly, N343 works as a gate control opening of the S1-RBD in going from the ‘down’ to ‘up’ state in the glycosylated spike (S) protein [41]. The structure was obtained from the CHARMM-GUI COVID-19 protein archive [42], corresponding to a full glycosylated, all-atom model of homotrimeric SARS-CoV-2 spike proteins in open conformation (PDB ID: 6VSB [43]). After selecting the FA2G2 glycan, it was connected to each S1-RBD by linking the reducing end of GlcNAc1 to the ND2 atom of the N343. Interestingly, the MD simulations show a reduced flexibility of the FA2G2 in Omicron S1-RBD in comparison to WT S1-RBD (compare Figure S11a,b with S11c). In the WT-S1-RBD, the octasaccharide equally populated two major conformations, defined by the $\chi_{1}$ dihedral angle around the N343 side chain, according to the following consecutive atoms:$\;C-C_{\alpha }-C_{\beta }-C_{\gamma }$. In fact, $\chi_{1}$ populates almost equally the *trans* and *gauche^+^* states, corresponding to approximate values of 160° and 70°, respectively (Figure 5a).

**Figure 6:**
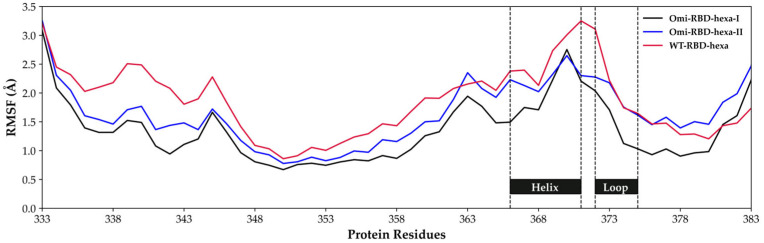
Zoom-in of the RMSF plot of Figure S9 centered on the amino acids between 333 and 383 and calculated on the meta-trajectories of Omi-S1-RBD-hexa-I, Omi-S1-RBD-hexa-II, and WT-S1-RBD-hexa. The helix 366–371 and the loop 372–375 are highlighted.

FA2G2 has a dual orientation; when $\chi_{1}$ populates the *trans* state, the glycan occupies a pocket close to the 366–371 helix and the 372–375 loop without making any contacts with the residues of site I, as shown by the glycan with green tubes in Figure 5c and supported by the heatmap in Figure S12. At values of $\chi_{1}$ around 70°, the glycan moves closer to site I, making contacts with both hexa and the residues of site I, primarily K356, as shown by the glycan with cyan tubes in Figure 5c (see also Figure S12). In this conformation, we observed partial detachment of hexa from site I, suggesting a competitive effect for site I between FA2G2 and hexa (see Video S1a). On the other hand, for Omi-S1-RBD-hexa-I and hexa-II systems (Figure 5b,d and Figure S13, respectively), the glycan sampled essentially the *trans* conformation $\chi_{1}$ (Figure 5b, Video S1b) and, consequently, it remained proximal to the 372–375 loop, leaving site I unperturbed, as shown by the green glycan in Figure 5d (see also Figure S12). Additionally, in a minor populated state, where $\chi_{1}$ is around 75° (populated less than 5%), the octasaccharide is solvent-exposed and loses contact with S1-RBD, as shown by the cyan glycan in Figure 5d (see also Figure S13). Notably, the reduced mobility observed for FA2G2 in the Omicron systems can be attributed to the G339D mutation. In the WT-S1-RBS, G339 is small and apolar, allowing free movements of the octasaccharide within the 366-371 helix to site I. In contrast, substitution with aspartic acid in the Omicron S1-RBD introduces a larger, negatively charged side chain that impairs movements toward site I. D339 is constantly in contact with the GlcNAc^1^, GlcNAc^2^, and Fuc^8^ units of FA2G2, impairing its movements (Figure S14). Moreover, in the Omicron simulations, interactions between FA2G2 and residues in the helix loop region 336–375 reduce the flexibility of this region, stabilizing the N-terminal part of the S1-RBD sequence compared to the wild-type simulations, as can be observed by comparing the RMSF of the corresponding residues in Figure 6.

In addition, S371L, S373P, and S375F mutations contribute to this stabilization. In fact, it has been demonstrated [44] that these mutated residues can establish a higher number of hydrogen bonds with helix 366–371, reinforcing the local structure as confirmed by the decreased RSMF values in Figure 6.

### 3.5. Selection of the Complexes Omi-S1-RBD-Hexa Using the Experimental STD_0_

The RedMat application [25] allows for the identification of geometries of the simulated complex that agree with selected experimental *STD_0_*. The application calculates a score (R-NOE factor) for each frame, according to Equation (2). This score is based on the standard deviation between each calculated *STD_0_* value $\left(STD^{calc,k}\right)$ and corresponding experimental value ($STD^{exp,k})$ considering selected *k*-signals. An R-NOE smaller than 0.3 normally indicates a good agreement between the experimental and theoretical proton binding epitopes.

(2) $$RNOE=\sqrt{\frac{\sum {\left(STD^{exp,k}-STD^{calc,k}\right)}^{2}}{\sum {\left(STD^{exp,k}\right)}^{2}}}$$

**Figure 7:**
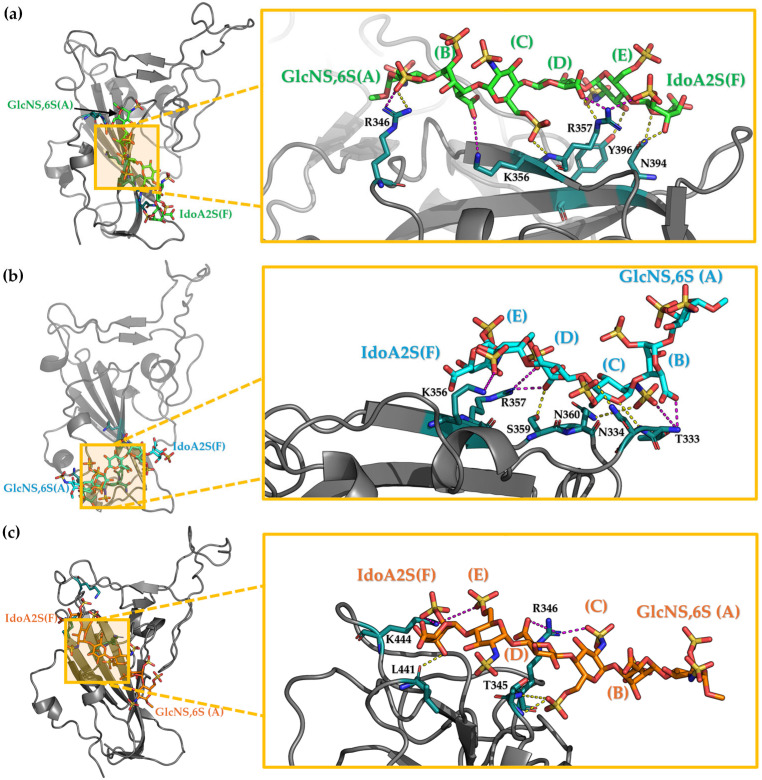
Representative structures of first and second clusters of Omi-S1-RBD-hexa-I (panels (**a**,**b**) and the first cluster of Omi-S1-RBD-hexa-II (panel (**c**)), which are characterized by the lowest R-NOE (R-NOE = 0.04, 0.11, 0.11, respectively). The ligands are illustrated as sticks: green and cyan for the carbon of two clusters of Omi-S1-RBD-hexa-I, while orange for the main populated cluster of Omi-S1-RBD-hexa-II. Red, yellow, and blue correspond to oxygen, sulfur, and nitrogen atoms, respectively. Each monosaccharide is labeled with its corresponding capital letter. The protein is shown as a gray cartoon. Residues involved in the interaction are represented as teal sticks, with magenta indicating salt bridges and yellow for hydrogen bonds. The oligosaccharide FA2G2 is bound to N343, and all the protons are omitted for clarity.

All the STD-NMR resonances of the central residues of hexa (from sugar E to B; Table S1) were selected for comparison with the corresponding theoretical signals. Terminal sugars A and F were excluded due to their high conformational flexibility (Figure 3). For both Omi-S1-RBD-hexa-I and Omi-S1-RBD-hexa-II systems, the comparison between experimental and theoretical STD binding epitopes reveals variation in the extent of agreement. Only 25% of the RBD-Omi-hexa-I and 18% of RBD-Omi-hexa-II present R-NOEs smaller than 0.3 (Figure S15). To identify the dominant binding modes that have been selected by RedMat, a cluster analysis was performed on the MD-simulated meta-trajectories of Omi-S1-RBD-hexa-I and Omi-S1-RBD-hexa-II, corresponding to the bound state condition (Section 2.1.2) and with R-NOE ≤ 0.3 (Table S5 and Figure S16). For each cluster, the hexa geometry that possesses the lowest R-NOE was selected as the representative member of the cluster and reported in Figure 7.

The cluster analysis applied to the Omi-S1-RBD-hexa-I system reveals the two most populated geometries, with populations of 43% and 39%, respectively (Table S5). These correspond to two distinct binding modes. In the first cluster (43%), hexa is positioned in the central part of site I (Figure 7a and Figure S16a). GlcNS6S(C) forms a hydrogen bond between the backbone of R357 and the C6-OSO_3_^−^ group, while the COO^−^ group of GlcA(D) forms a salt bridge with R357. In the second cluster (39%), hexa is in a region proximal to site I, forming interactions with residues S359 and N360 (Figure 7b and Figure S16a). In this case, the C6-OSO_3_^−^ group of glucosamine C engages in a hydrogen bond with N334, and the N-sulfo group forms hydrogen bonds with T333 and N360. Additionally, the COO^−^ group of GlcA(D) forms a salt bridge with R357 and a hydrogen bond with S359.

In Omi-RBD-hexa-II, two representative clusters were identified with populations of 73% and 10%. The most populated are reported in Figure 7c (see also Figure S15b and Table S5). The ligand, whose position is consistent with that of the original docking pose, preserves residue IdoA2S(F) oriented toward the ACE region, in hydrogen bond contact with the backbone of L441 [IdoA2S-3-O-H --- O=C-L441] and ionic interactions with K444. By contrast, the N-sulfo group of sugar C and the carboxyl group of sugar D establish ionic interactions with R346, while the C6-OSO_3_^−^ group of sugar C forms additional hydrogen bonds with NH of the backbone of T345.

In summary, the interaction between hexa and Omi-S1-RBD presents different binding modes that agree with the observed *STD_0_* signals, in which the central sugars C and D are preferentially in contact with S1-RBD. These binding modes are reported in Figure 7a–c and indicate a low level of specificity of the interaction, as was previously figured out by Parafioriti et al. [18]. In all of these complexes, characterized by R-NOE < 0.3, the main interactions involve glucosamine C and glucuronic acid D, although contributions from glucosamine E and iduronic acid F are also observed. In contrast, units A and B are frequently solvent-exposed (Omi-RBD-hexa-II, Figure 7c), which accounts for their lower *STD_0_* values (Table S1).

**Figure 8:**
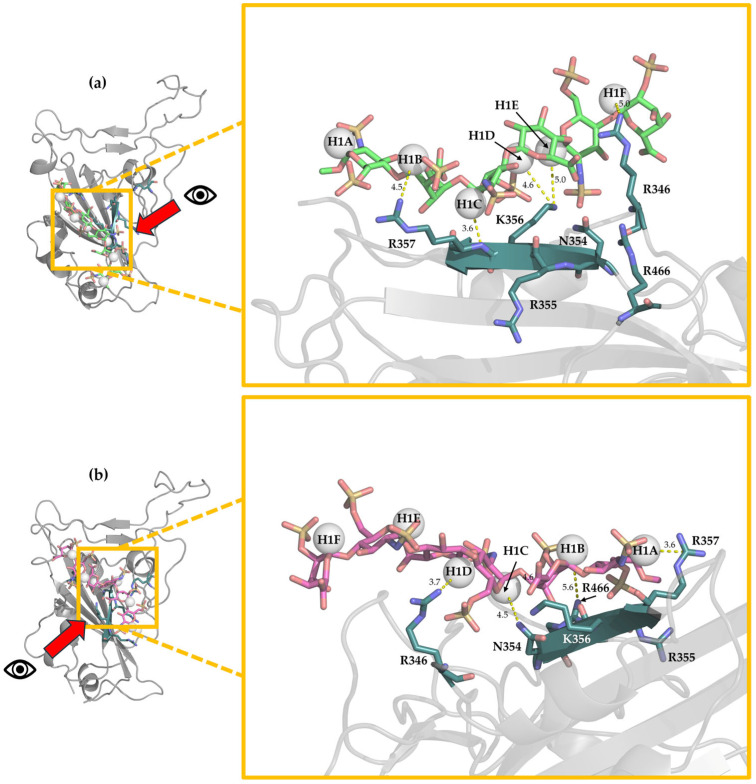
Cluster I and cluster II (panel (**a**) and panel (**b**), respectively) of WT-S1-RBD-hexa with enlargement of the binding region. Hexa is illustrated as sticks: green and magenta for the carbon of cluster I and II of WT-S1-RBD-hexa-I. Red, yellow, and blue correspond to oxygen, sulfur, and nitrogen atoms, respectively. The S1-RBD is represented as white ribbon, while residues involved in the interaction (R346, N354, R355, K356, R357, and R466) are represented as sticks (teal for carbon, red for oxygen, and blue for nitrogen) and labeled. The anomeric protons are labeled, and the distance from the closest S1-RBD residue is reported.

The cluster analysis of the WT-S1-RBD-hexa system that was applied to the meta-trajectory filtered only with the bound state condition (Section 2.1.2) reveals two representative clusters (Figure 8a,b and Table S5c). The most populated (48%) shows the ligand bound to site I (Figure 8a). In this binding mode, residues GlcNS6S(A) and IdoA2S(B) interact with R357, while GlcNS6S(C) and GlcA(D) form electrostatic contacts with K356. Additionally, GlcNS6S(E) and IdoA2S(F) establish interactions with R346 and R345. In the less populated cluster (20%), the terminal residues from IdoA2S(F) and GlcNS6S(E) are solvent-exposed, whereas residues from A to D are located in the upper part of site I (Figure 8b), establishing electrostatic interactions with R357 and R466, K356 and R466, and K356 and R346, respectively. Both clusters are consistent with our previous study [18]. Notably, the distance between all anomeric protons and the protein surface is less than 5 Å, indicating that all residues are likely to contribute to binding, and the saturation transfer from the protein to the ligand is efficient, supporting, qualitatively, the STD intensities observed previously [18].

## 4. Conclusions

Heparan sulfate (HS) plays an important role in SARS-CoV-2 infection by promoting the active (open) conformation of the spike protein and acting as a receptor for viral particles at the cell surface. The more infectious variants, such as Omicron, appear to have undergone selection involving an increase in the overall positive charge of the spike protein, which suggests potentially enhanced binding for both the ACE2 and HS receptors. In fact, the mutations observed in the Omicron S1-RBD are localized predominantly to the ACE2 binding interface and do not alter the HS binding region significantly.

We characterized the interaction of a hexasaccharide (representing short chains of heparan sulfate) with the Omicron S1-RBD, using a combination of multiple independent MD simulations and STD-NMR. The oligosaccharide presents a relatively stiff conformation in unbound and bound states with both Omicron as well as WT-S1-RBDs. The hexa oligosaccharide explores the shallow cavity corresponding to the site I of these S1-RBDs, which is identified by the following adjacent polar or positively charged side chains: R346, N354, R355, K356, R357, and R466. This glycan undergoes partial detachment, re-binding, alternating rotation and translation movements, probing site I and the surrounding area without reaching the ACE2 binding region.

To experimentally support the 3D model of such low-affinity glycan-S1-RBD complexes, the RedMat application was used. This computational workflow identified three principal modes of interaction between hexa and the Omicron S1-RBD, indicating that the recognition event is characterized by a low degree of specificity. As has been observed for the wild-type strain [18], the glycan binds site I using both of its major surfaces, particularly involving the central glucosamine C and glucuronic acid D residues, while the terminal residues A, B, D, and F display greater variability and solvent exposure.

In the WT-RBD-hexa complex, clustering of the bound frames revealed interactions extending across the entire ligand; all sugar units were positioned within 5 Å of the protein surface.

Interestingly, WT S1-RBD and its Omicron variant present distinct conformational behavior of the biantennary N-glycosyl FA2G2 at N343, which correlates with a different ratio of *trans/gauche^+^* states of the dihedral angle $\chi_{1}$. This corresponds to the distinct capacity of this glycan to shield site I of S1-RBD and/or detach the bound hexa. In the wild-type system, the FA2G2 glycan exhibits higher mobility, corresponding to a comparable population of *trans* and *gauche^+^* states of $\chi_{1}$. In this condition, FA2G2 shields the site I of S1-RBD, thereby interfering with the interaction between hexa and S1-RBD. In contrast, the G339D mutation in the Omicron S1-RBD confines the FA2G2 between helix 366–371 and loop 372–375. In this condition, $\chi_{1}$ populates its *trans* state, stabilizing the S1-RBD structure, which is further reinforced by the point mutations S371L, S373P, and S375F. It is important to underline that the conformational flexibility of the FA2G2 glycan, summarized by $\chi_{1}\;$, can be slightly overestimated since this study is based on a limited sub-system of the full glycosylated trimeric spike (S) protein; therefore, all the perturbative effects arising from the subunits that in the trimeric spike protein surround the S1-RBD are disregarded. Consequently, the conformations sampled by FA2G2, as well as its ability to perturb the site I, differ slightly in free S1-RBD in comparison to the corresponding structure when, in up conformation, is part of the spike S trimer.

We can summarize that the multivalent interaction observed in the Omicron S1-RBD, reflected by the presence of multiple binding modes and the reduced perturbative effect that the FA2G2 glycan moiety applies to site I of the Omicron S1-RBD, may enhance the accessibility of this latest S1-RBD to cellular HS, potentially supporting the increased affinity that the Omicron S1-RBD exhibits in comparison to the WT S1-RBD towards HS oligosaccharides. Finally, we underline that these multivalent and low-specificity aspects of the interactions between the HS oligosaccharides and S1-RBD of SARS-CoV-2 require a succession of uninterrupted positive patches, Arg, Lys, and His (at suitable pH) in site I, which are accessible on the surface of the viral receptor by a sticky (polyanion) double-face HS oligosaccharide. These aspects, while crucial to dissect how the intermolecular forces are distributed between the molecular surfaces that become in contact and drive the cell surface—viral receptor recognition—involved a somewhat oversimplified model: the forefront ‘tip’ of spike protein and an HS hexasaccharide. Therefore, rigorously, these behaviors cannot be directly generalized to a more extended system in which the whole S1 subunit of the trimeric spike protein is surrounded and may bind long HS chains, as is supposed to happen when SARS-CoV-2 approaches and explores the surface of the target cell in search of the most efficient way to enter.

## Data Availability

The data presented in this study are available upon request from the corresponding author.

## Supplementary Materials

The following supporting information can be downloaded at: https://www.mdpi.com/article/10.3390/biom15091343/s1, Figure S1: Top-ranked poses of hexa and S1-RBD protein of Omicron docking (Omi-S1-RBD-hexa-I on panel a, Omi-S1-RBD-hexa-II on panel b) and Wild-type docking (WT-S1-RBD-hexa on panel c); Figure S2: (a) ^1^H-STD-NMR build-up curves of hexa using all the saturation times (0.5, 1, 2, 3, 5s). (b) Enlargement of ^1^H-13C HSQC-dept of hexa - OmicronS1-RBD, with superimposition of ^1^H-STD NMR spectrum acquired with a saturation time of 3s; Figure S3: The 3D structure of hexa with the anomeric and C6 protons represented as white spheres; Figure S4: Putative heparin binding sites in S1-RBD protein (magenta for site I, orange for site II and green for site III) and red for residues S359 and N360; Figure S5: Stoddart diagram of Cremer – People parameters of Omi-S1-RBD-hexa-I, (a), Omi-S1-RBD-hexa-II, (b) and WT-S1-RBD-hexa (c) and hexa in the unbound state (d) metatrajectory; Figure S6: Scatter plots of inter-glycosidic dihedral angles *ϕ**_i_*/*ψ**_i_* for each replica of Omi-S1-RBD-hexa-I (a,b), Omi-S1-RBD-hexa-II (c,d) and WT-S1-RBD-hexa (e,f), and hexa in unbound state (g,h); Figure S7: Root Mean Square Deviation (RMSD) of hexa (top row), calculated for all the ring heavy atoms and interlinkage oxygen atoms upon fitting the backbone atoms of the S1-RBD. RMSD of the backbone of S1-RBD (bottom row); Figure S8: Root means square fluctuation (RMSF) of the backbone (Cα) of S1-RBD calculated from the MD simulation meta-trajectories of Omi-S1-RBD-hexa-I (black line), Omi-S1-RBD-hexa-II (blue line), and WT-S1-RBD-hexa (red line) (a); Figure S9: Graphical representation of the shallow and concave topology of the WT-S1-RBD Site I binding site; Figure S10: Distance plot between the centre of mass of GlcA(D) (red line) and GlcNS,6S (C) (blue line) and the centre of mass of the nearest neighbouring residue of RBD protein for each replica of Omi-S1-RBD-hexa-I (a), Omi-S1-RBD-hexa-II (b) and WT-S1-RBD-hexa (c); Figure S11: Graphical representation of the conformation populated by the FA2G2 glycan at N343 in Omi-S1-RBD-hexa-I (a), Omi-S1-RBD-hexa-II (b), and WT-S1-RBD-hexa (c); Figure S12: Heatmap of contacts observed between hexa and FA2G2 and between FA2G2 and S1-RBD in WT-S1-RBD-hexa (a,b) and Omi-S1-RBD-hexa-I (**c**) and RBD-Omi-hexa-II (d) meta-trajectories; Figure S13: Conformational analysis of FA2G2 in Omi-S1-RBD-hexa-I (red), Omi-S1-RBD-hexa-II (green) and WT-S1-RBD-hexa (blue) concatenated simulations; Figure S14: The G339D mutation in Omicron S1-RBD reduces FA2G2 glycan flexibility; Figure S15: Evolution of the R-NOE factor for RBD-Omi-hexa-I (a) and RBD-Omi-hexa-II (b) over the metatrajectory; Figure S16: Representative clusters of Omi-S1-RBD (a) and Omi-S1-RBD-hexa-II (b) and WT-S1-RBD-hexa (c) are depicted as stick using different colours as reported in the legend; Table S1: Absolute STD values for each NMR experiment and relative STD percentages (STD_0_ %) of selected protons of the hexa; Table S2: Ring conformers population, calculated on the combined trajectory of the metatrajectory of RDB-Omi-hexa-I (a), RDB-Omi-hexa-II (b), RDB-WT-hexa (c) and hexa in the unbound state (d); Table S3: Average *ϕ**_i_*/*ψ**_i_* values of each inter-glycosidic bond of the hexa in the free (Free hexa) and bound states; Table S4: Results of contact analysis for Omi-S1-RBD-hexa-I (tables a and b for salt bridges and hydrogen bonds), Omi-S1-RBD-hexa-II (tables c and d for salt bridges and hydrogen bonds), and WT-S1-RBD-hexa (tables e and f for salt bridges and hydrogen bonds); Table S5. Cluster analysis result of Omi-S1-RBD-hexa-I (table a) and Omi-S1-RBD-hexa-II (table b) and WT-S1-RBD-hexa (table c), reporting the members and the relative percentage (perc. %) for the first five families determined; Video S1: During the production run, the FA2G2 octasaccharide adopted the gauche⁺ conformational state.

## Abbreviations

The following abbreviations are used in this manuscript:

ACE2	Angiotensin-Converting Enzyme 2
GlcA	Glucuronic Acid
GlcNS,6S	Glucosamine, N-, 6-O-Disulfated
HS	Heparan Sulfate
IdoA2S	Iduronic Acid 2-O-Sulfated
MD	Molecular Dynamics
RBD	Receptor-Binding Domain
RMSD	Root Mean Square Deviation
RMSF	Root Mean Square Fluctuation
SARS	Severe Acute Respiratory Syndrome
STD-NMR	Saturation Transfer Difference Nuclear Magnetic Resonance
