# Supplementary material for: Interaction Between Heparan Sulfate Oligosaccharide and the Receptor-Binding Domain of the Wild-Type and Omicron Variant of the SARS-CoV-2 Spike Protein
Source: Biomolecules. 2025 Sep 19;15(9):1343. doi: 10.3390/biom15091343 (PMC12467571; doi:10.3390/biom15091343)
Supplement: Supplementary file 1 [file biomolecules-15-01343-s001.zip › biomolecules-3853263-supplementary.pdf]

Supplementary Materials

Interaction between Heparan Sulfate oligosaccharide and the receptor binding domain of Wuhan and Omicron variants of the SARS-CoV-2 spike protein

Marco Mandalari [1,2], Michela Parafioriti [2], Minghong Ni [2], Francesca Benevelli [3], Monica Civera [1,*], Stefano Elli [2,*] and Marco Guerrini [2]

[1] Dipartimento di Chimica, Università degli Studi di Milano, Via Golgi 19, 20133 Milan, Italy; marco.mandalari@unimi.it (M.M.)
[2] Istituto di Ricerche Chimiche e Biochimiche 'G. Ronzoni', via Giuseppe Colombo 81, 20133 Milan, Italy; mandalari@ronzoni.it (M.M); parafioriti@ronzoni.it (M.P.); niminghong@ronzoni.it (M.H.N.); guerrini@ronzoni.it (M.G.)
[3] BioSpin Business Unit, Bruker Italia S.r.l., Viale Vincenzo Lancetti 43, 20158 Milan, Italy; francesca.benevelli@bruker.com (F.B.)
* Correspondence: monica.civera@unimi.it (M.C.); elli@ronzoni.it (S.E.)

[a] Università degli Studi di Milano, Dipartimento di Chimica, Via Golgi 19 20133 Milan, Italy.
[b] Istituto di Ricerche Chimiche e Biochimiche 'G. Ronzoni via Giuseppe Colombo 81, 20133 Milan, Italy.
[c] BioSpin Business Unit, Bruker Italia S.r.l. , Viale Vincenzo Lancetti, 43, 20158 Milan, Italy.
* These are the corresponding authors.

Movie S1a. Movie S1a. WT-S1–RBD–hexa.

During the production run, the FA2G2 octasaccharide adopted the gauche$^+$ conformational state. In this state, the glycan is oriented towards the binding site, exerting a competitive binding effect on the hexa ligand and leading to its partial detachment from site I. The protein is shown as a white cartoon, while the key residues of the binding site are represented as sticks (teal for carbon, red for oxygen, and blue for nitrogen). The FA2G2 octasaccharide and the hexa oligosaccharide are also shown as sticks, using the same colour code (cyan [FA2G2] or green [hexa], red for oxygen, blue for nitrogen, and yellow for sulfur).

Movie S1b. Omi-S1–RBD–hexa-I.

During the production run, the FA2G2 octasaccharide adopted only the trans conformational state. The D339G mutation in Omicron S1–RBD, introducing a bulkier and negatively charged residue, prevents rotation of FA2G2 into the gauche$^+$ state. Instead, the octasaccharide is oriented towards helix 366–371 and loop 372–375, further stabilising the structure, which is already stiffened by mutations S371L, S373P, and S375. The protein is shown as a white cartoon, while the key residues of the binding site are represented as sticks (teal for carbon, red for oxygen, and blue for nitrogen). The FA2G2 octasaccharide and the hexa oligosaccharide are also shown as sticks, using the colour code (cyan [FA2G2] or green [hexa], red for oxygen, blue for nitrogen, and yellow for sulfur).

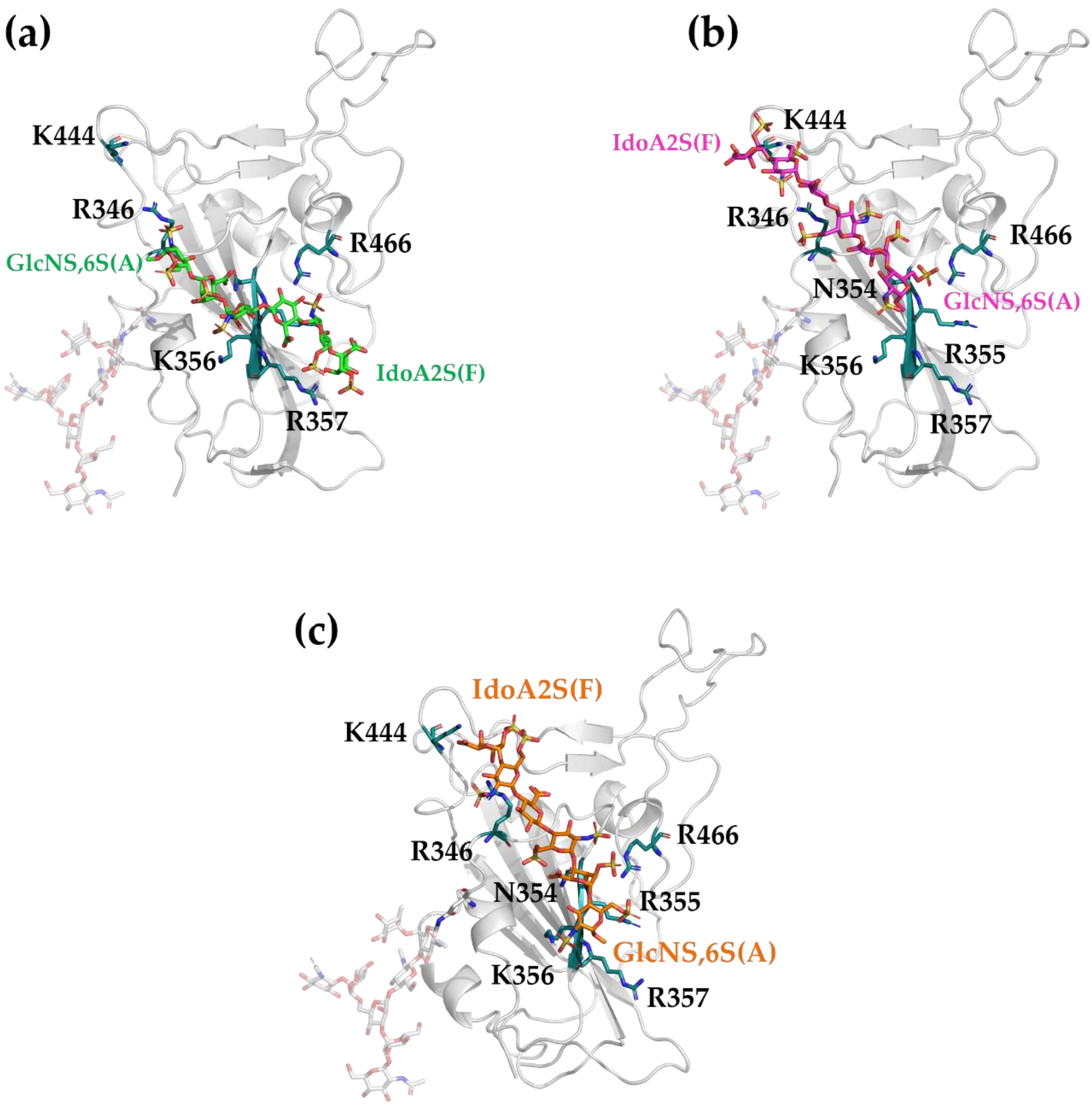

Figure S1. Top-ranked poses of hexa and S1-RBD protein of Omicron docking (Omi-RBD-hexa-I on panel a, Omi-RBD-hexa-II on panel b) and Wild-type docking (WT-RBD-hexa on panel c). A grey cartoon represents the viral protein while the hexa is depicted by a stick model (carbon in green, magenta and orange, respectively; oxygen in red, nitrogen in blue, and sulfur in yellow). Binding site key residues (R346, N354, R355, K356, R357, K444 and R466) are labelled and coloured as teal sticks. The reducing and non-reducing end are labelled as GlcNS,6S(A) and IdoA2S(F), respectively.

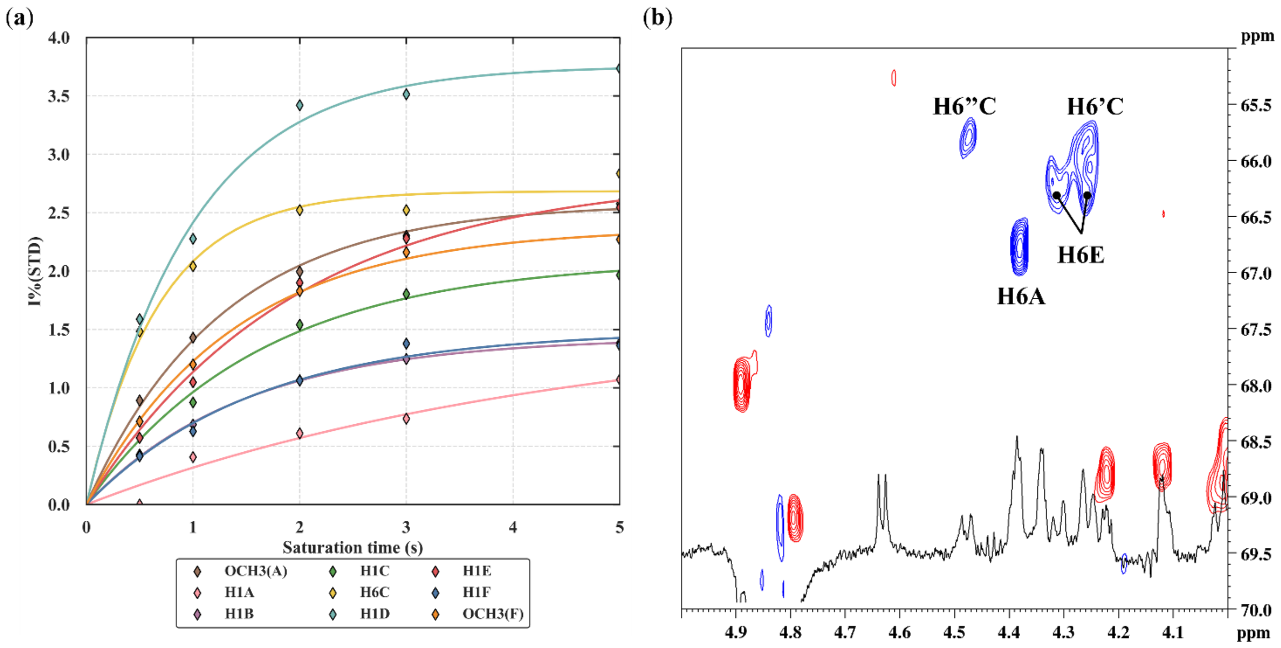

Figure S2. **(a)** ^{1}H-STD-NMR build-up curves of hexa using all the saturation times (0.5, 1, 2, 3, 5s). **(b)** Enlargement of ^{1}H-^{13}C HSQC-dept of hexa - OmicronS1-RBD, with superimposition of ^{1}H-STD NMR spectrum acquired with a saturation time of 3s.

Table S1. Absolute STD values for each NMR experiment and relative STD percentages (STD_0 %) of selected protons of the hexa.

	Absolute STD %					Relative STD_0 %
**Sat. Time(s)**	**0.5**	**1**	**2**	**3**	**5**	
**OCH_3(A)**	0.89	1.43	2.00	2.30	2.58	51%
**H1A**	0.00	0.41	0.61	0.74	1.07	9%
**H1B**	0.43	0.69	1.06	1.25	1.38	24%
**H1C**	0.57	0.87	1.54	1.80	1.97	32%
**H6C**	1.48	2.04	2.52	2.52	2.84	100%
**H1D**	1.59	2.28	3.42	3.51	3.74	96%
**H1E**	0.57	1.05	1.90	2.28	2.55	36%
**H1F**	0.41	0.63	1.06	1.38	1.36	23%
**OCH_3(F)**	0.71	1.20	1.83	2.16	2.27	43%

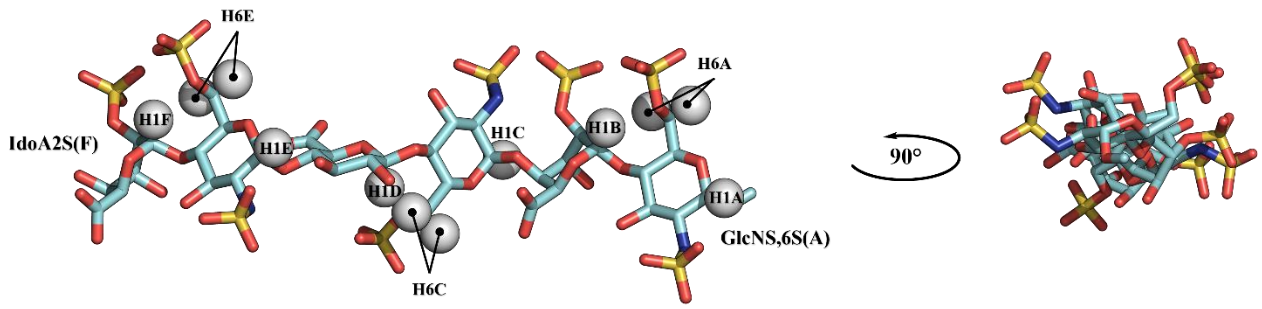

Figure S3. The 3D structure of hexa with the anomeric and C6 protons represented as white spheres. The negative electrostatic repulsion among charged groups causes glucosamine E, C, and A to orient the 6-OSO_3^- groups in the opposite direction along the polysaccharide. Additionally, the ligand has a linear arrangement of sulfated groups along its molecular axis, forming two primary interaction faces.

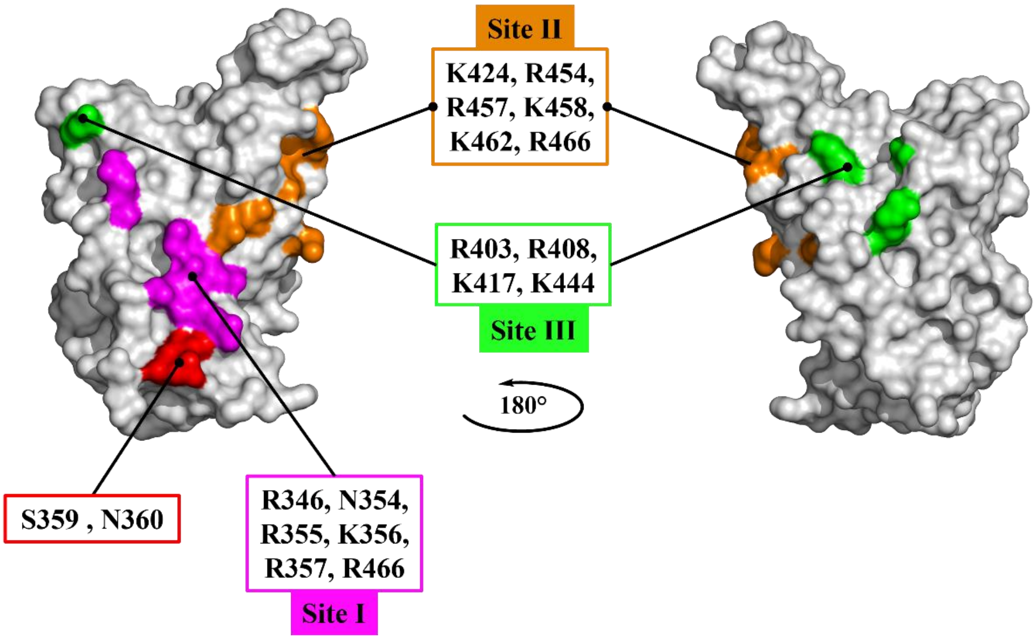

Figure S4. Putative heparin binding sites in S1-RBD protein (magenta for site I, orange for site II and green for site III) and red for residues S359 and N360.

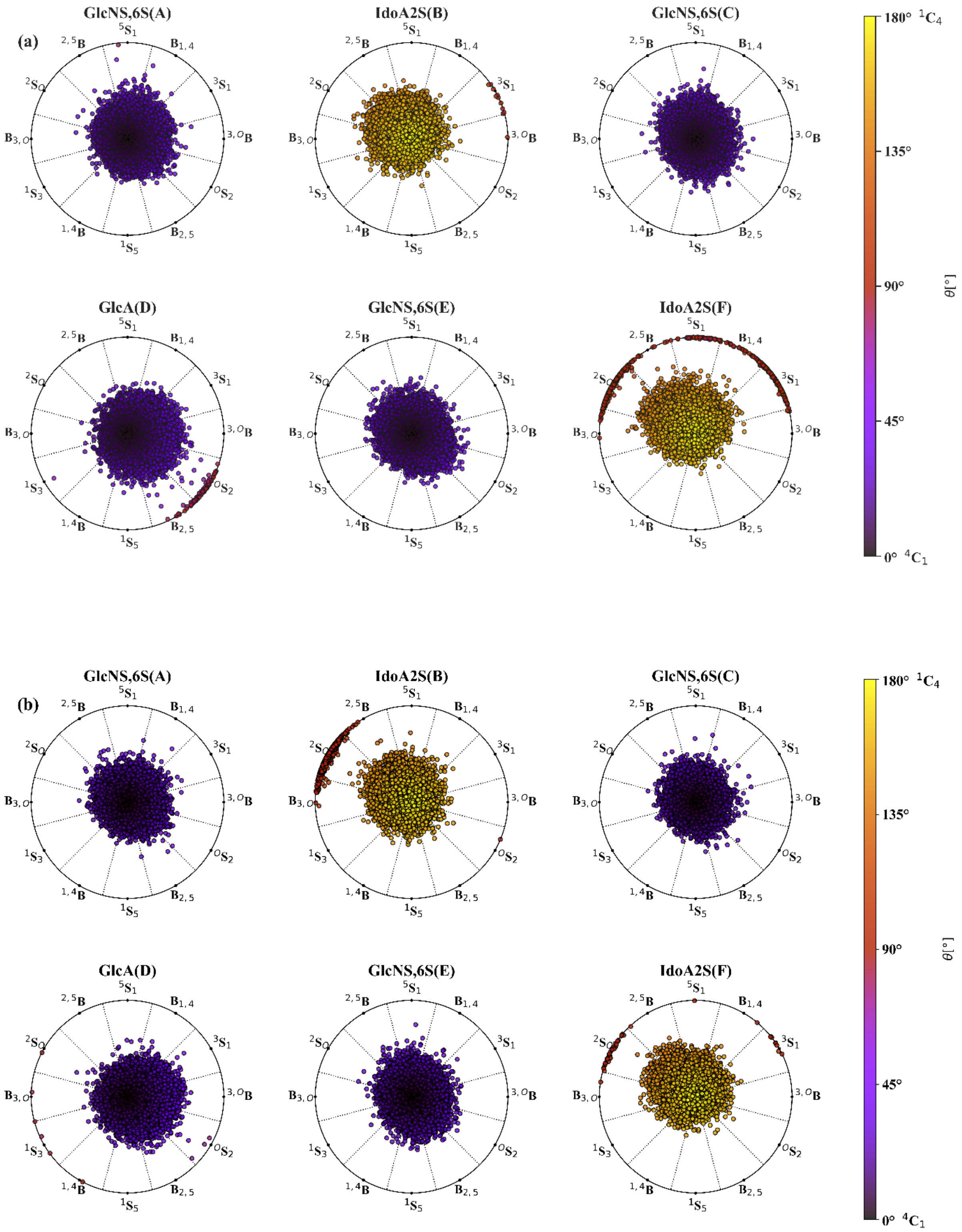
(a)
GlcNS,6S(A)
IdoA2S(B)
GlcNS,6S(C)
GlcA(D)
GlcNS,6S(E)
IdoA2S(F)
(b)
GlcNS,6S(A)
IdoA2S(B)
GlcNS,6S(C)
GlcA(D)
GlcNS,6S(E)
IdoA2S(F)
$^{5}S_{1}$
$B_{1,4}$
$^{3}S_{1}$
$^{3,O}B$
$^{O}S_{2}$
$B_{2,5}$
$^{1}S_{5}$
$^{1,4}B$
$^{1}S_{3}$
$B_{3,O}$
$^{2}S_{O}$
$^{2,5}B$
180° $^{1}C_{4}$
135°
90°
45°
0° $^{4}C_{1}$
θ[°]

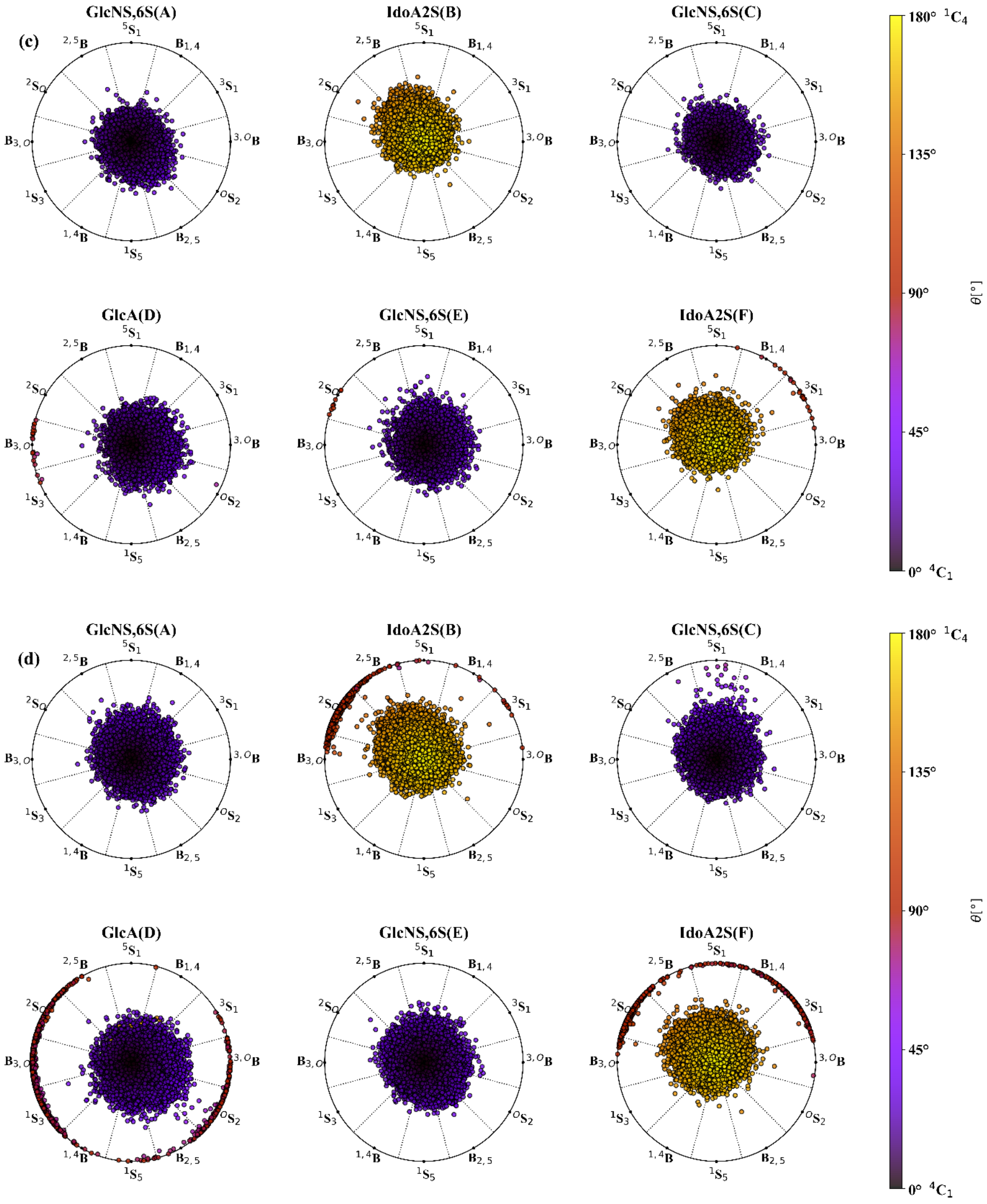

Figure S5. Stoddart diagram of Cremer – People parameters of Omi-S1-RBD-hexa-I, (a), Omi-S1-RBD-hexa-II, (b) and WT-S1-RBD-hexa (c) and hexa in the unbound state (d) metatrajectory. The colour code identifies the position of each ring conformation on the corresponding spherical polar representation: violet $^{4}C_{1}$ conformation (θ = 0°), yellow for the $^{1}C_{4}$ (θ = 180°) conformation, and red for boat (B) and skew-boat (S) conformation (θ = 90°).

Table S2. Ring conformers population, calculated on the combined trajectory of the meta-trajectory of RDB-Omi-hexa-I (a), RDB-Omi-hexa-II (b), RDB-WT-hexa (c) and hexa in the unbound state (d). Each population was labelled using the basic conformations in the IUPAC nomenclature: the two chairs $^4C_1$ and $^1C_4$ are at the sphere's poles (corresponding to θ = 0° or 180°, respectively). Instead, the 6 boats (B) and skew-boats (S) conformers are located at the equator (θ=90°) population, each of which is characterised by a different value of ϕ. Conformations populated at less than 0.5% are omitted, and the percentages are rounded to the nearest whole number.

(a)	RBD-Omi-hexa-I – Ring Conformers (%)				
	$^4C_1$	$^2S_0$	$B_{3,O}$	$^1S_3$	$^1C_4$
**GlcNS,6S(A)**	100	-	-	-	-
**IdoA2S(B)**	-	-	-	-	100
**GlcNS,6S(C)**	100	-	-	-	-
**GlcA(D)**	100	-	-	-	-
**GlcNS,6S(E)**	100	-	-	-	-
**IdoA2S(F)**	-	1	1	-	98

(b)	RBD-Omi-hexa-II – Ring Conformers (%)				
	$^4C_1$	$^2S_0$	$^3S_1$	$^0S_2$	$^1C_4$
**GlcNS,6S(A)**	100	-	-	-	-
**IdoA2S(B)**	-	6	-	-	94
**GlcNS,6S(C)**	100	-	-	-	-
**GlcA(D)**	100	-	-	-	-
**GlcNS,6S(E)**	100	-	-	-	-
**IdoA2S(F)**	-	-	-	-	100

(c)	RBD-WT-hexa – Ring Conformers (%)				
	$^4C_1$	$^2S_0$	$^3S_1$	$^0S_2$	$^1C_4$
**GlcNS,6S(A)**	100	-	-	-	-
**IdoA2S(B)**	-	-	-	-	100
**GlcNS,6S(C)**	100	-	-	-	-
**GlcA(D)**	100	-	-	-	-
**GlcNS,6S(E)**	100	-	-	-	-
**IdoA2S(F)**	-	-	-	-	100

(d)	Hexa in free state – Ring Conformers (%)				
	$^4C_1$	$^2S_0$	$^3S_1$	$^0S_2$	$^1C_4$
**GlcNS,6S(A)**	100	-	-	-	-
**IdoA2S(B)**	-	3	-	-	97
**GlcNS,6S(C)**	100	-	-	-	-
**GlcA(D)**	94	1	3	1	1
**GlcNS,6S(E)**	100	-	-	-	-
**IdoA2S(F)**	-	1	-	-	99

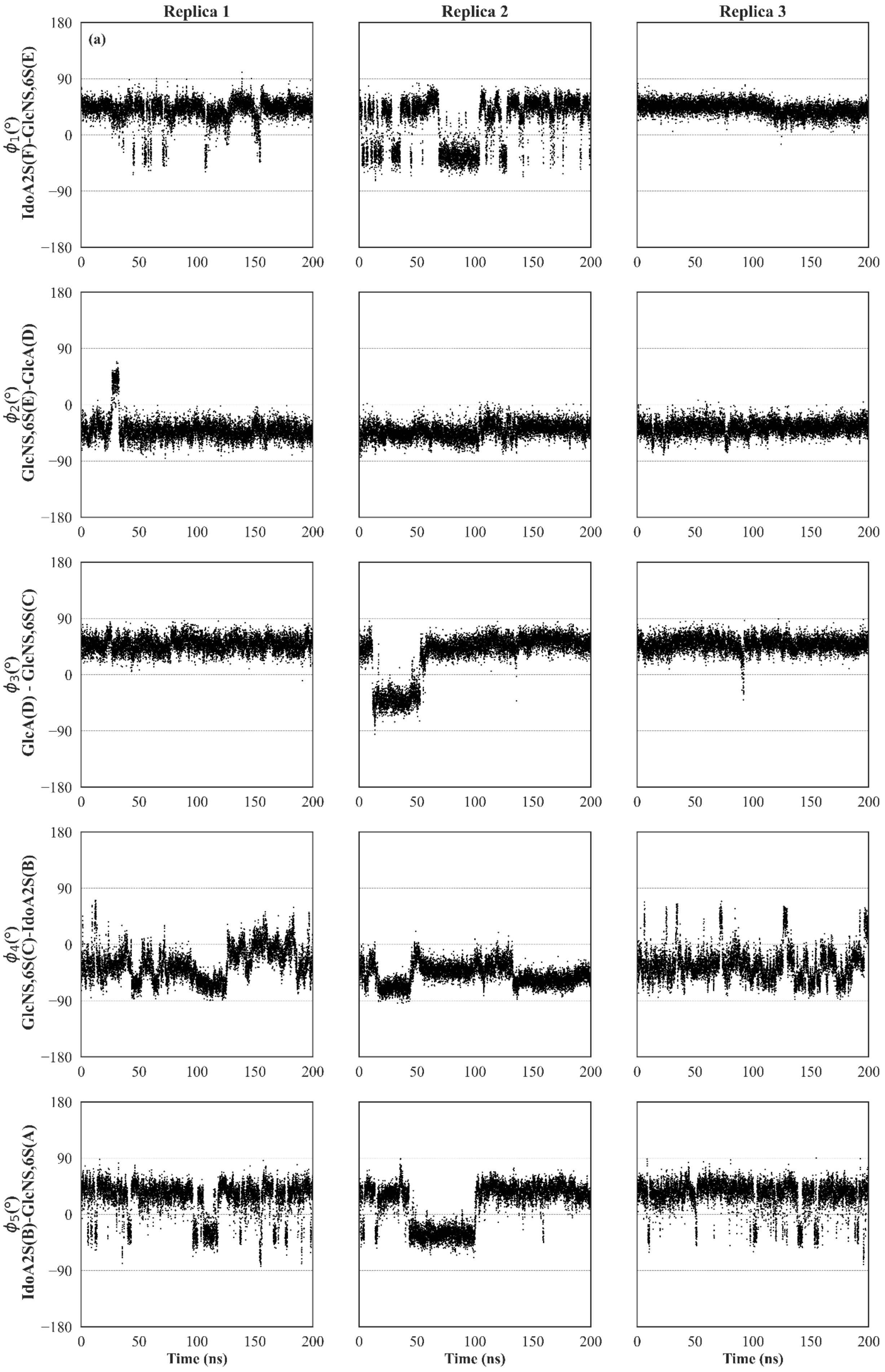
Replica 1
Replica 2
Replica 3
(a)
ϕ_1(°)
IdoA2S(F)-GlcNS,6S(E)
ϕ_2(°)
GlcNS,6S(E)-GlcA(D)
ϕ_3(°)
GlcA(D) - GlcNS,6S(C)
ϕ_4(°)
GlcNS,6S(C)-IdoA2S(B)
ϕ_5(°)
IdoA2S(B)-GlcNS,6S(A)
Time (ns)

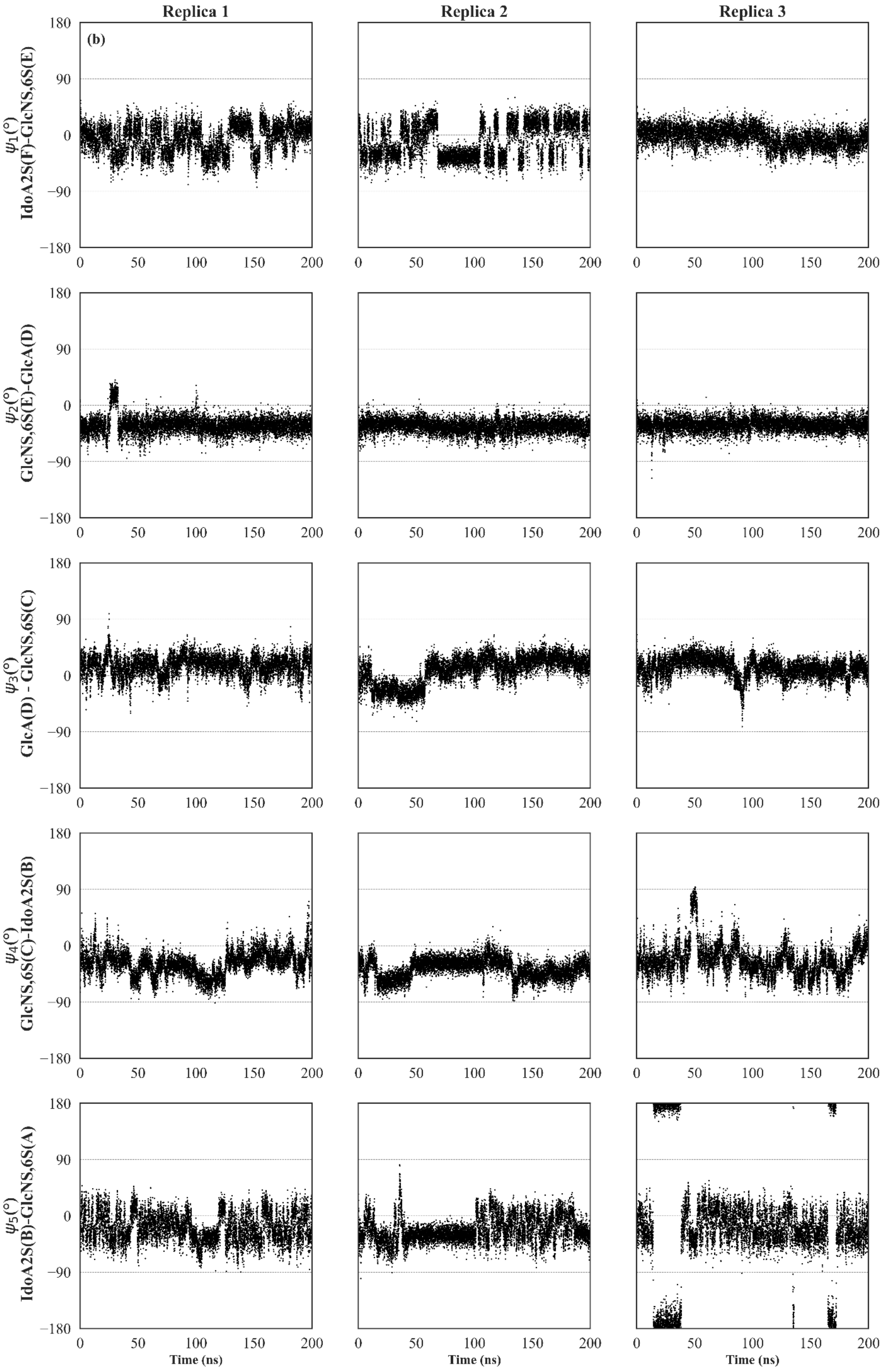

Replica 1
Replica 2
Replica 3
(b)
ψ1(°) IdoA2S(F)-GlcNS,6S(E)
ψ2(°) GlcNS,6S(E)-GlcA(D)
ψ3(°) GlcA(D) - GlcNS,6S(C)
ψ4(°) GlcNS,6S(C)-IdoA2S(B)
ψ5(°) IdoA2S(B)-GlcNS,6S(A)
Time (ns)

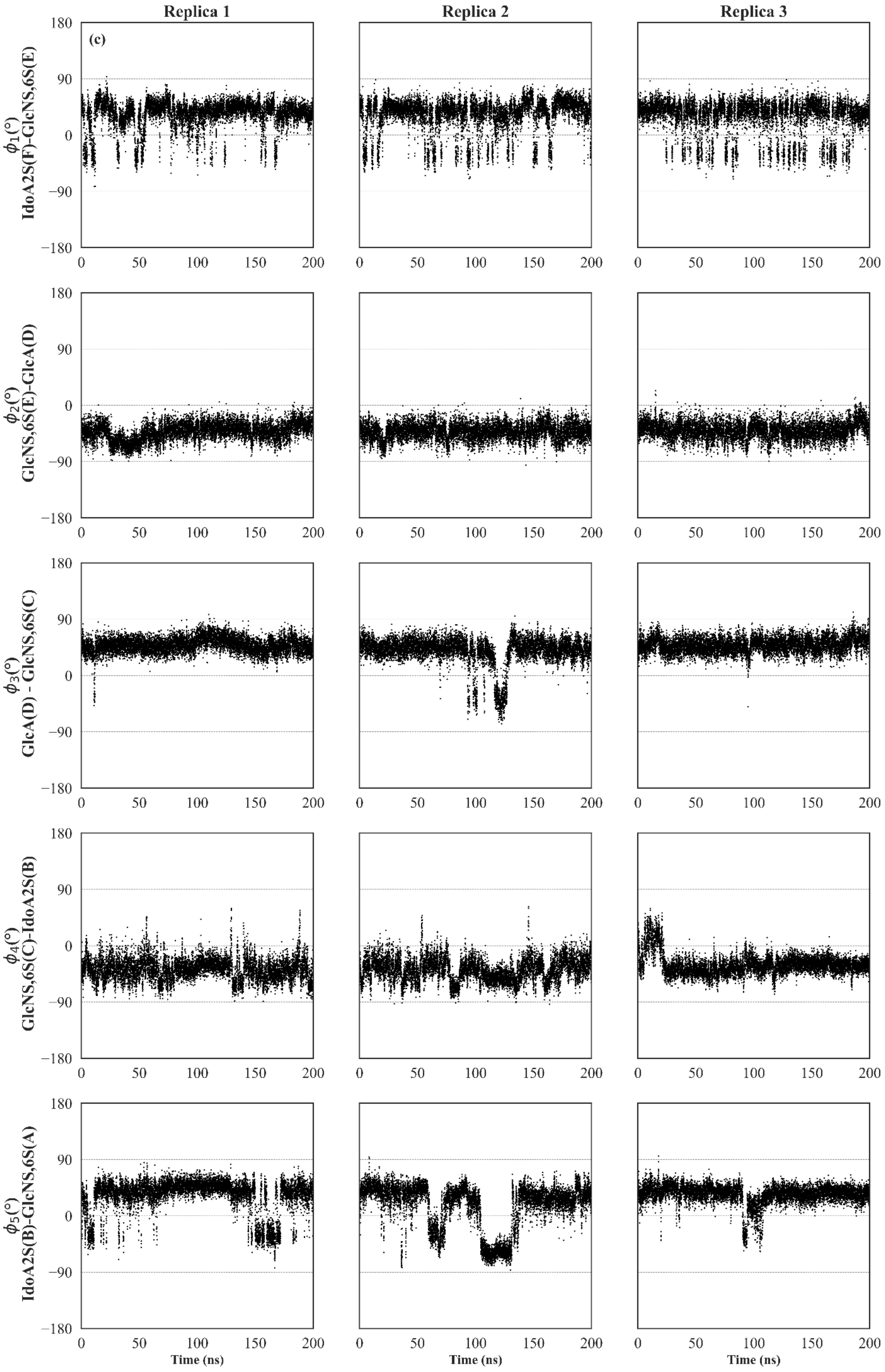

Replica 1
Replica 2
Replica 3
(c)
φ1(°) IdoA2S(F)-GlcNS,6S(E)
φ2(°) GlcNS,6S(E)-GlcA(D)
φ3(°) GlcA(D) - GlcNS,6S(C)
φ4(°) GlcNS,6S(C)-IdoA2S(B)
φ5(°) IdoA2S(B)-GlcNS,6S(A)
180
90
0
−90
−180
0
50
100
150
200
Time (ns)

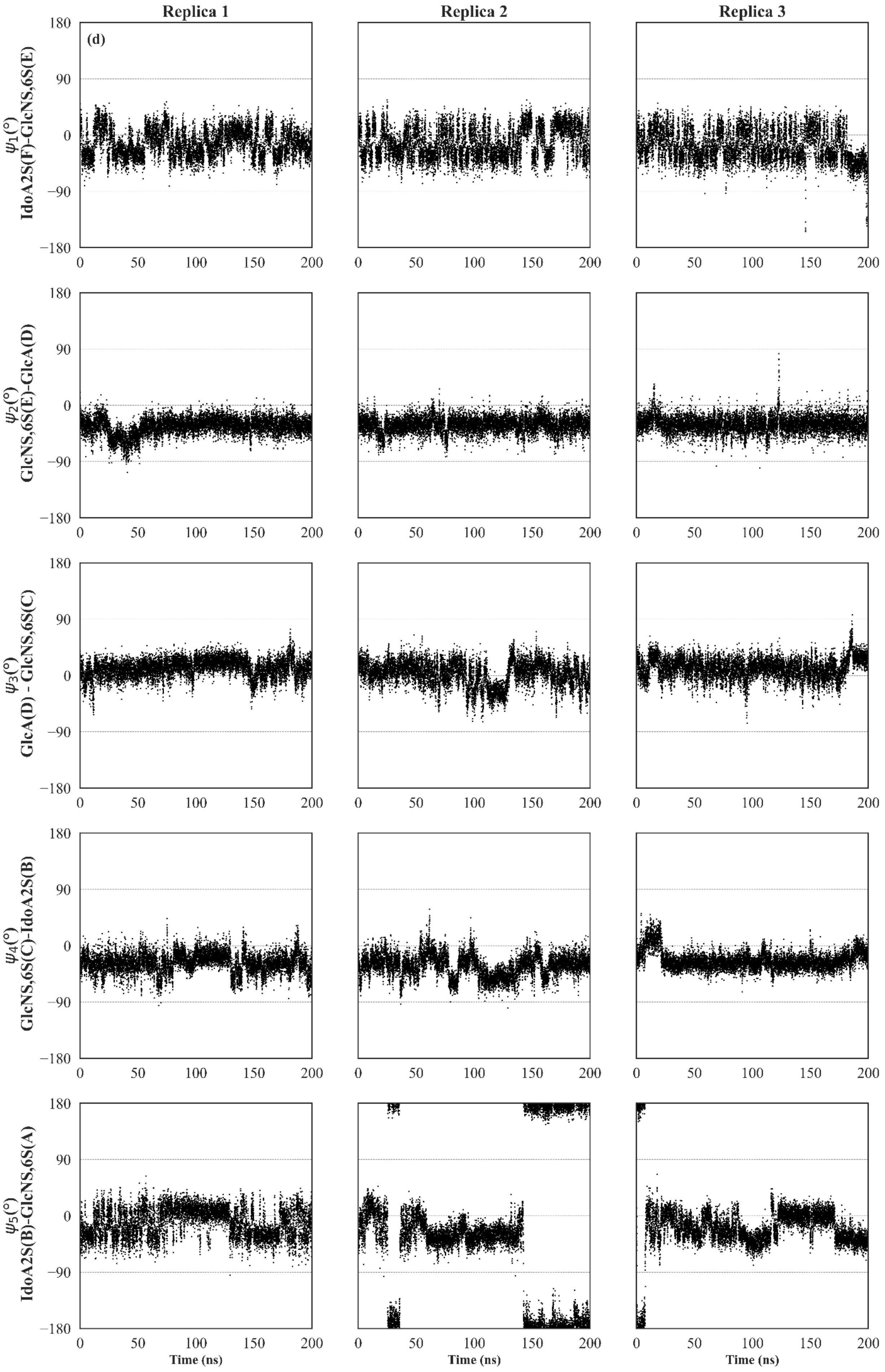

Replica 1
Replica 2
Replica 3
(d)
ψ_1(°) IdoA2S(F)-GlcNS,6S(E)
ψ_2(°) GlcNS,6S(E)-GlcA(D)
ψ_3(°) GlcA(D) - GlcNS,6S(C)
ψ_4(°) GlcNS,6S(C)-IdoA2S(B)
ψ_5(°) IdoA2S(B)-GlcNS,6S(A)
180
90
0
−90
−180
0
50
100
150
200
Time (ns)

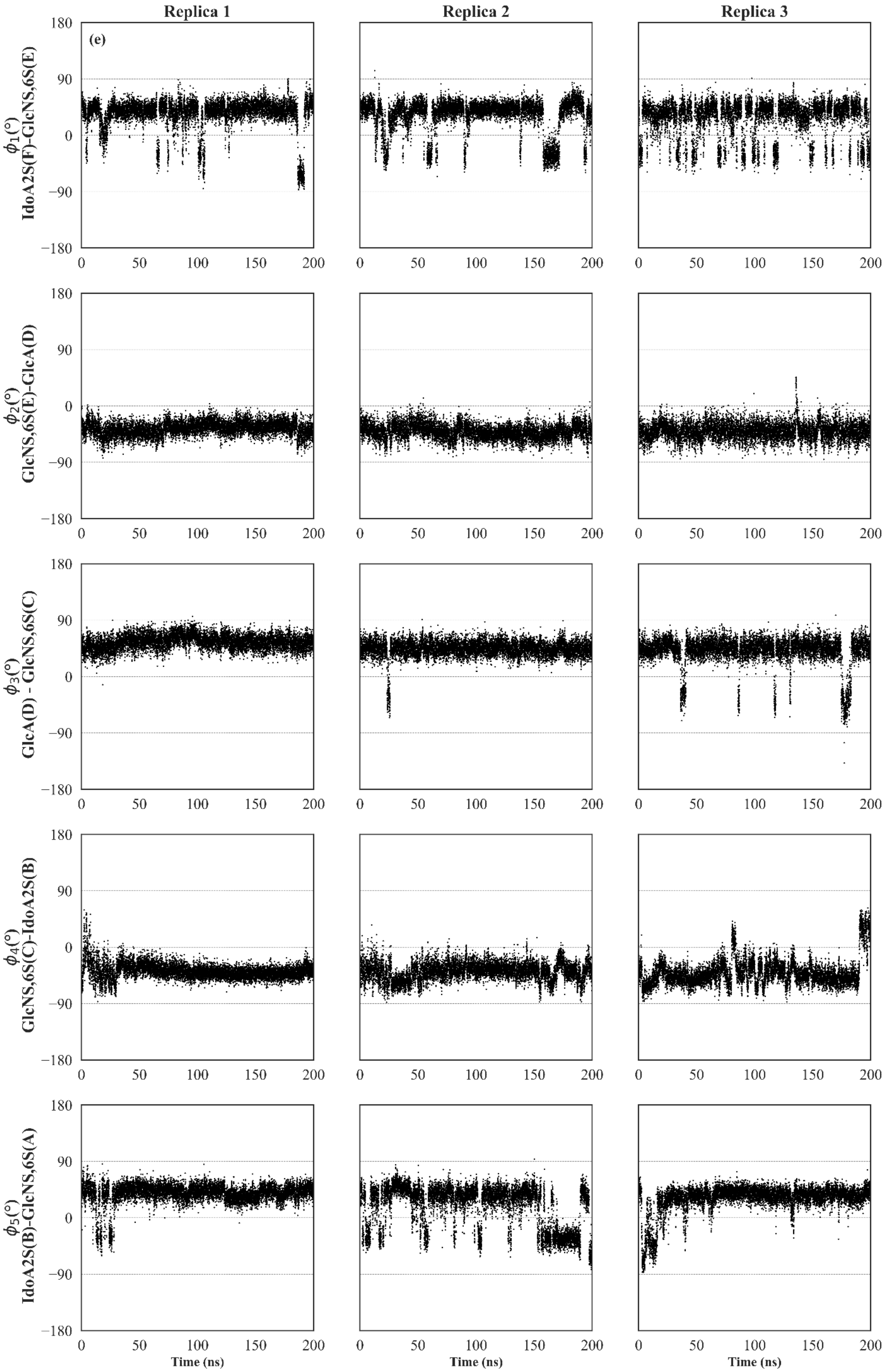

Replica 1
Replica 2
Replica 3
(e)
ϕ_1(°) IdoA2S(F)-GlcNS,6S(E)
ϕ_2(°) GlcNS,6S(E)-GlcA(D)
ϕ_3(°) GlcA(D) - GlcNS,6S(C)
ϕ_4(°) GlcNS,6S(C)-IdoA2S(B)
ϕ_5(°) IdoA2S(B)-GlcNS,6S(A)
180
90
0
−90
−180
0
50
100
150
200
Time (ns)

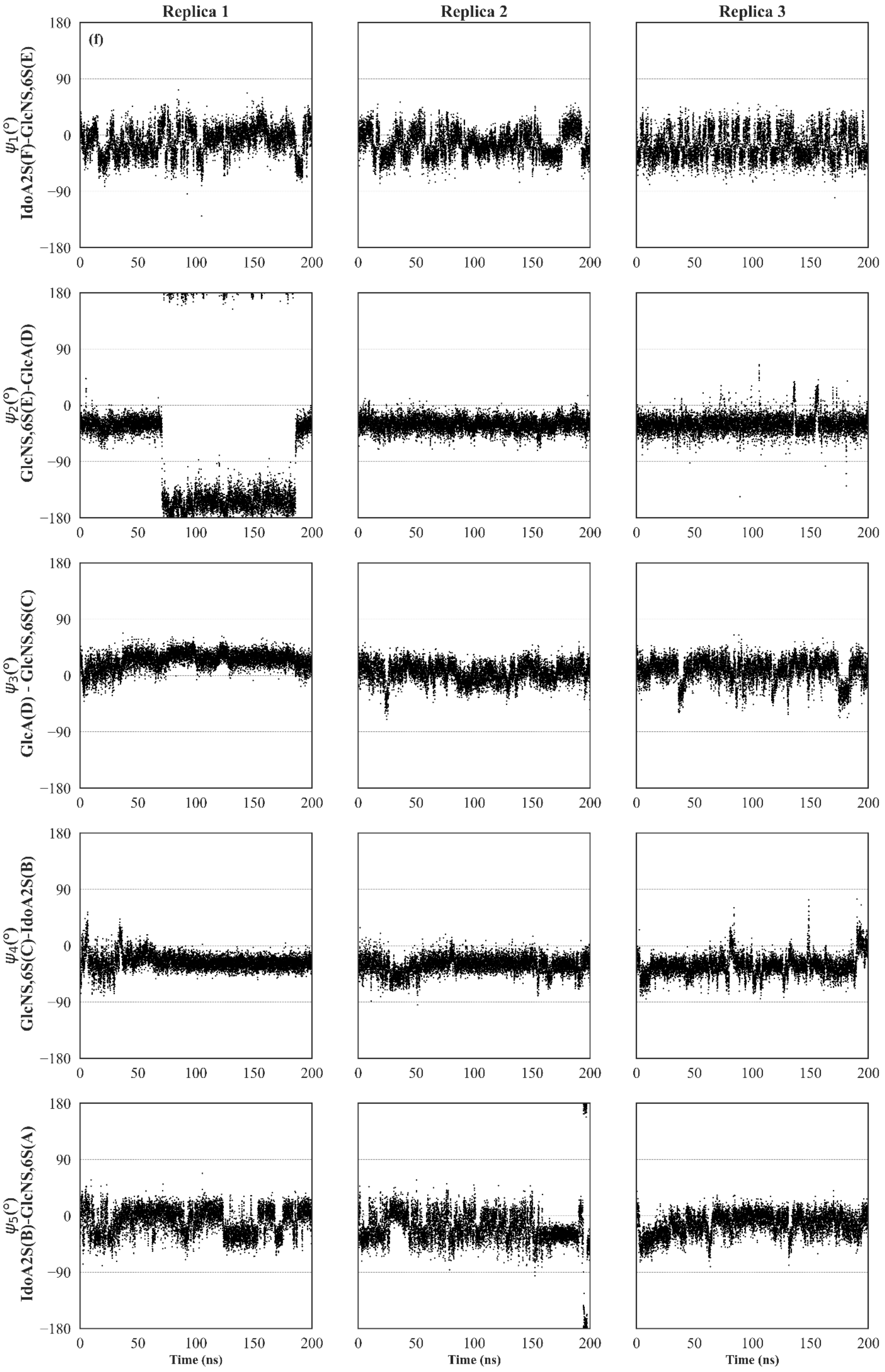

Replica 1
Replica 2
Replica 3
(f)
ψ1(°) IdoA2S(F)-GlcNS,6S(E)
ψ2(°) GlcNS,6S(E)-GlcA(D)
ψ3(°) GlcA(D) - GlcNS,6S(C)
ψ4(°) GlcNS,6S(C)-IdoA2S(B)
ψ5(°) IdoA2S(B)-GlcNS,6S(A)
180
90
0
−90
−180
0
50
100
150
200
Time (ns)

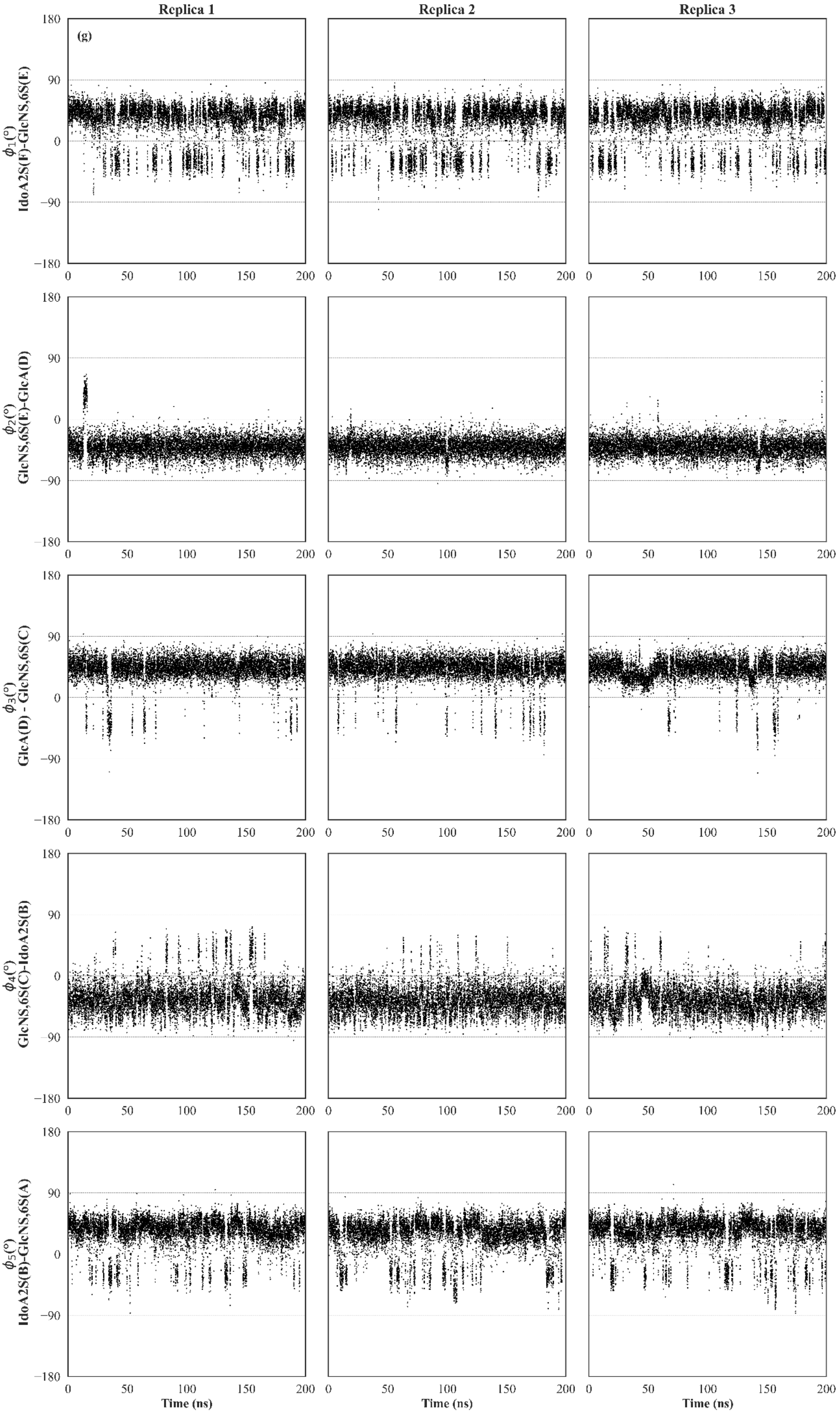

Replica 1
Replica 2
Replica 3
(g)
ϕ_1(°) IdoA2S(F)-GlcNS,6S(E)
ϕ_2(°) GlcNS,6S(E)-GlcA(D)
ϕ_3(°) GlcA(D) - GlcNS,6S(C)
ϕ_4(°) GlcNS,6S(C)-IdoA2S(B)
ϕ_5(°) IdoA2S(B)-GlcNS,6S(A)
Time (ns)

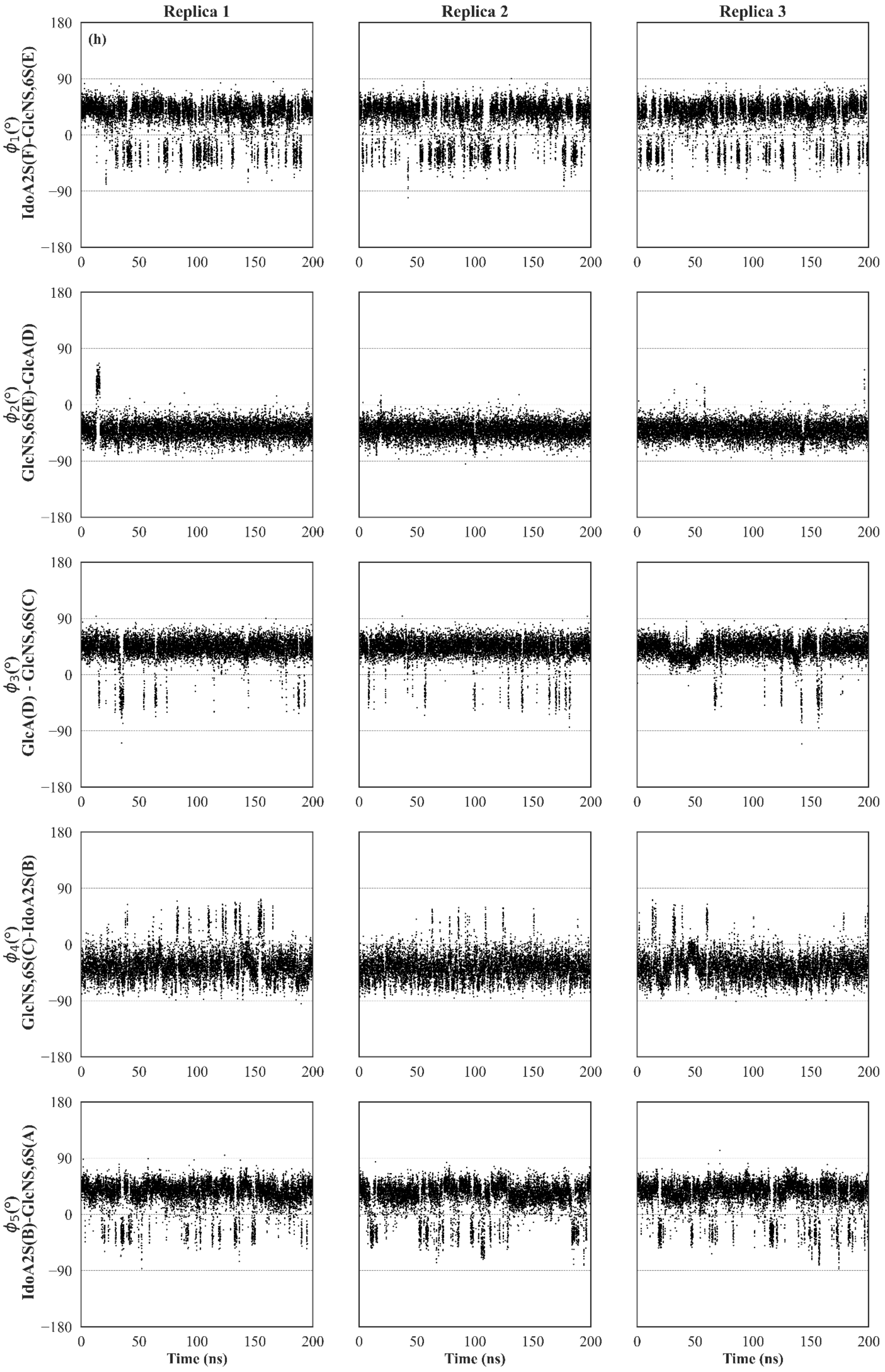

Replica 1
Replica 2
Replica 3
(h)
ϕ_1(°) IdoA2S(F)-GlcNS,6S(E)
ϕ_2(°) GlcNS,6S(E)-GlcA(D)
ϕ_3(°) GlcA(D) - GlcNS,6S(C)
ϕ_4(°) GlcNS,6S(C)-IdoA2S(B)
ϕ_5(°) IdoA2S(B)-GlcNS,6S(A)
180
90
0
−90
−180
0
50
100
150
200
Time (ns)

Figure S6 Scatter plots of inter-glycosidic dihedral angles ϕ_i/ψ_i for each replica of Omi-RBD-Hexa-I (a, b), Omi-RBD-Hexa-II (c, d) and WT-RBD-hexa (e, f), and hexa in unbound state (g, h).

Table S3. Average ϕ_i/ψ_i values of each inter-glycosidic bond of the hexa in the free (Free hexa) and bound states. Bound state values were calculated from the meta-trajectories filtered by the 'bound state' condition. The flexibility of the terminal glycosidic linkage between residue F and E, and between B and A, is greater than the central linkages E-D, D-C, C-B, as shown by wider peaks of ϕ_i/ψ_i distribution and for the presence of alternative weakly populated states.

Dihedral angle	Input angle (°)	Free hexa	Omi-S1-RBD-hexa-I	Omi-S1-RBD-hexa-II	WT-S1-RBD-hexa
$\phi_1 - \psi_1$	41 / 14	47 / 8	47 / 8	47 / 6	42 / -1
$\phi_2 - \psi_2$	-40 / -30	-40 / -28	-38 / -30	-40 / -30	-40 / -30
$\phi_3 - \psi_3$	60 / 30	45 / 13	50 / 21	47 / 11	55 / 25
$\phi_4 - \psi_4$	-39 / -33	-35 / -28	-40 / -28	-35 / -25	-42 / -28
$\phi_5 - \psi_5$	41 / 14	45 / -1	33 / -28	45 / 1	45 / 4

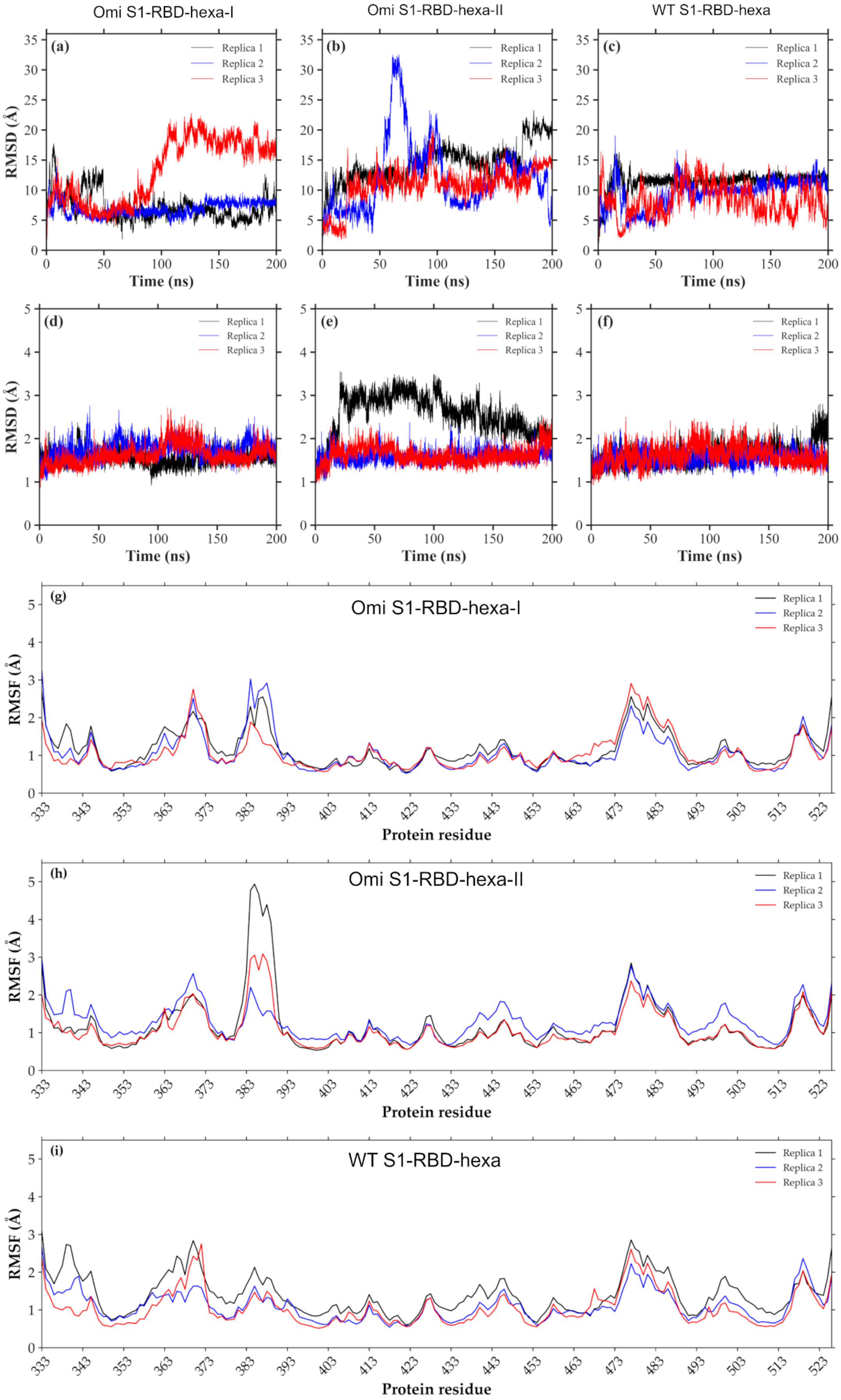
Omi S1-RBD-hexa-I
Omi S1-RBD-hexa-II
WT S1-RBD-hexa
(a)
(b)
(c)
(d)
(e)
(f)
Replica 1
Replica 2
Replica 3
RMSD (Å)
Time (ns)
(g)
Omi S1-RBD-hexa-I
(h)
Omi S1-RBD-hexa-II
(i)
WT S1-RBD-hexa
RMSF (Å)
Protein residue

Figure S7. Root Mean Square Deviation (RMSD) of hexa (panels a to c), calculated for all the ring heavy atoms and interlinkage oxygen atoms upon fitting the backbone atoms of the S1-RBD. RMSD of the backbone of S1-RBD (panels d to f). These RMSDs were calculated for the three MD simulation replicas of Omi-S1-RBD-hexa-I (a and d), Omi-S1-RBD-hexa-II (b and e), and WT-S1-RBD-hexa (panel c and f). Root Mean Square Fluctuation (RMSF) plotted vs residues of the complexes: Omi-S1-RBD-hexa-I (g), Omi-S1-RBD-hexa-II (h), and WT-S1-RBD-hexa (i). In all these panels the black, blue, and red lines indicate the replica 1, 2 and 3, respectively.

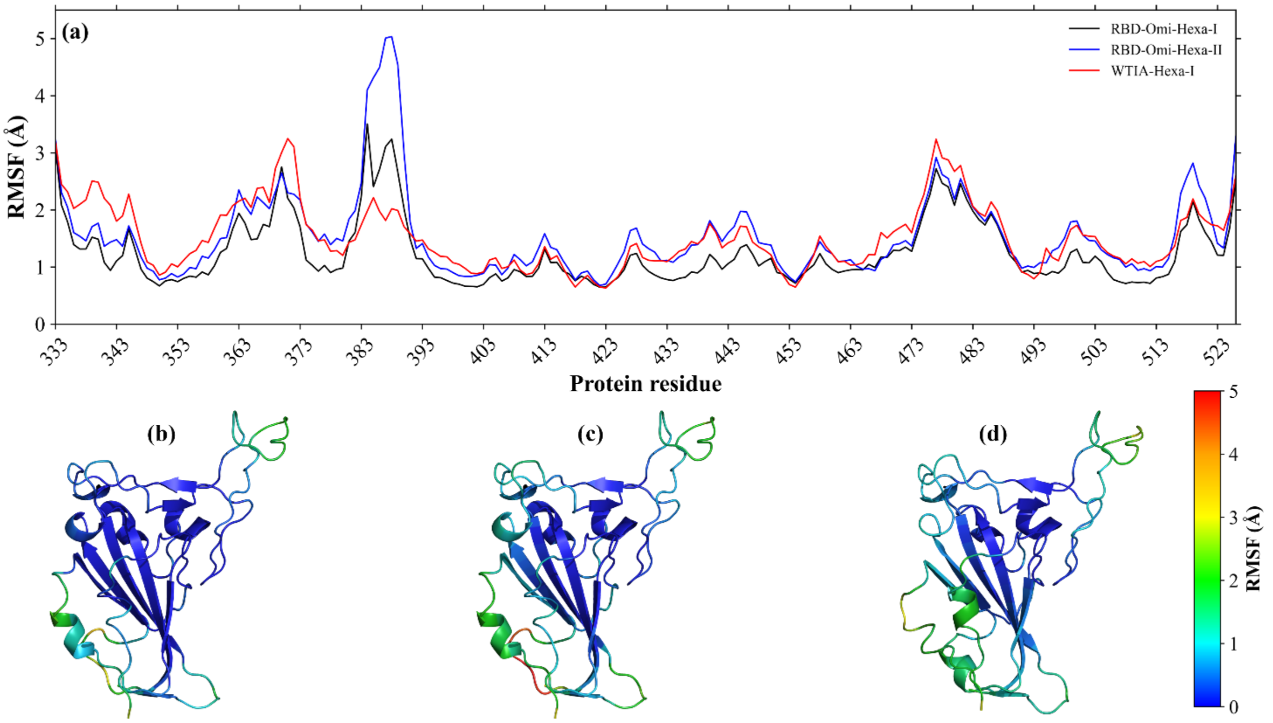

Figure S8. Root means square fluctuation (RMSF) of the backbone (Cα) of S1-RBD calculated from the MD simulation meta-trajectories of Omi-S1-RBD-hexa-I (black line), Omi-S1-RBD-hexa-II (blue line), and WT-S1-RBD-hexa (red line) (a). The corresponding Cα-RMSF values for Omi-S1-RBD-hexa-I (b), Omi-S1-RBD-hexa-II (c), and WT-S1-RBD-hexa (d) are mapped onto the RBD structure, shown as a cartoon representation. (b–d) Each residue is coloured according to its RMSF value.

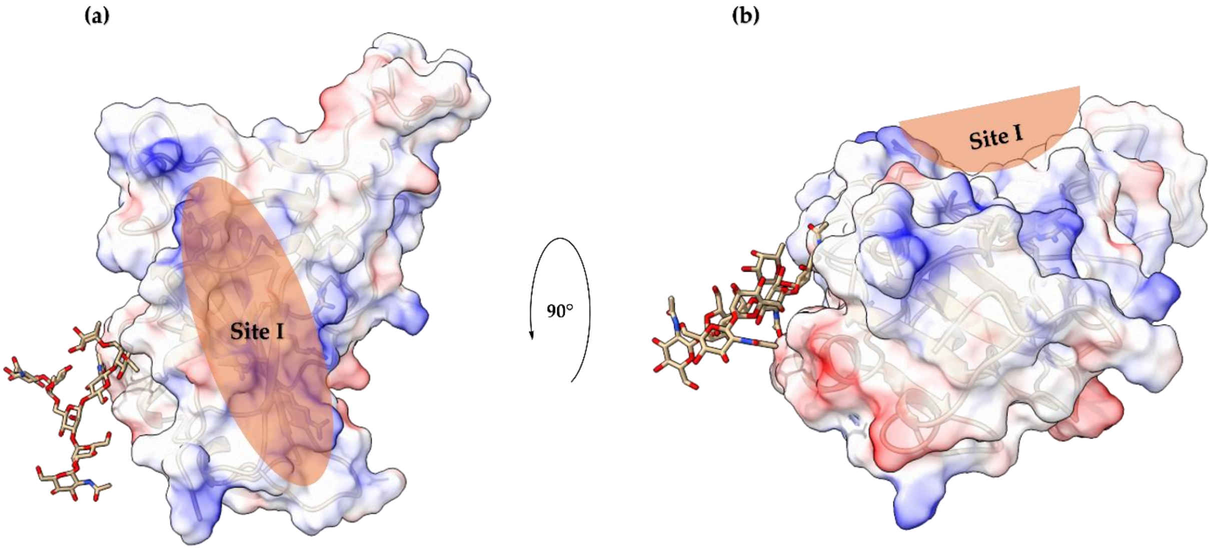

Figure S9. Graphical representation of the shallow and concave topology of the WT-S1-RBD Site I binding site. (a) Surface view of the WT-S1-RBD, with Site I highlighted as an orange oval. (b) Close-up view of the binding site, showing the shallow, concave pocket (highlighted in orange), suggesting limited depth and a potential preference for planar ligands.

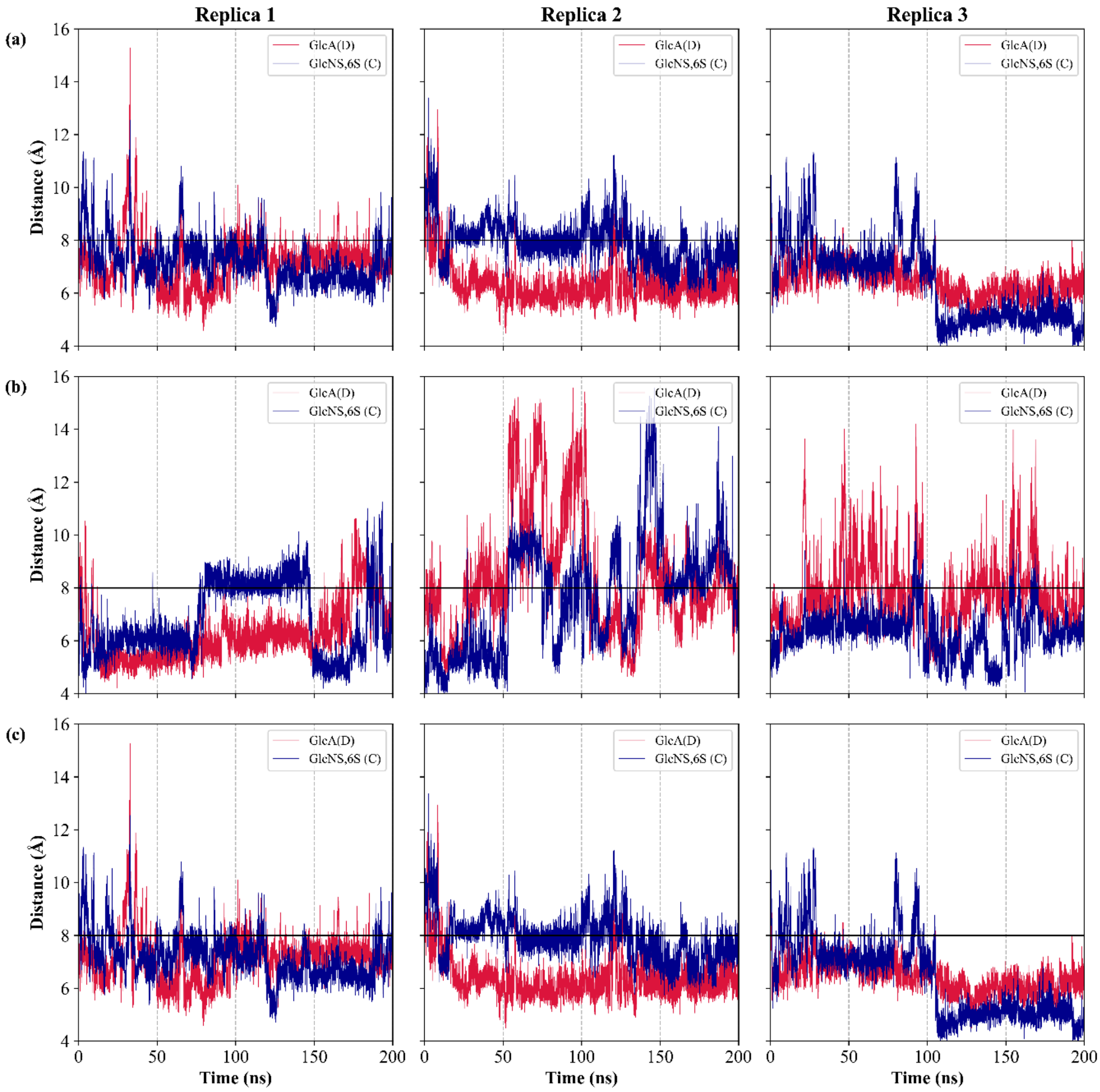

Figure S10. Distance plot between the centre of mass of GlcA(D) (red line) and GlcNS,6S (C) (blue line) and the centre of mass of the nearest neighbouring residue of RBD protein for each replica of Omi-S1-RBD-hexa-I (a), Omi-S1-RBD-hexa-II (b) and WT-RBD-hexa (c). The cutoff of 8Å is reported as a black line

Table S4. Results of contact analysis for Omi-RBD-hexa-I (tables a and b for salt bridges and hydrogen bonds), Omi-RBD-hexa-II (tables c and d for salt bridges and hydrogen bonds), and WT-RBD-hexa (tables e and f for salt bridges and hydrogen bonds). The salt bridges are computed based on the heavy atoms of the negatively charged group of hexa (C6 for COO-, S1 for -OSO3- or NHSO3-) and the heavy atoms of the positively charged RBD residues (NZ and NE for Lys; CZ and NE for Arg), with a cutoff set to 6Å. Conversely, hydrogen bonds are determined using the heavy atoms of the negatively charged group of hexa (C6 for COO-, S1 for -OSO3- or NHSO3-, and O3 for 3-OH) and the heavy atoms of RBD residues (ND2 for Asn, OG and N for Thr, N for Arg and Lys, OH for Tyr, OG for Ser), with a cutoff of 4.5Å. Contacts below 10% are excluded.

Table a

Protein Residue	Atom name	Ligand residue	Atom name	Percentage (%)	Distance (A°)	Dev. Std (A°)
R357	CZ	GlcA(D)- COO^-	C6	66.5	4.52	0.35
R357	NE	GlcA(D)- COO^-	C6	62.2	4.89	0.82
R357	CZ	GlcNS,6S(E) - 6 - OSO_3^-	S1	56.2	4.84	0.52
R357	NE	GlcNS,6S(E) - 6 - OSO_3^-	S1	40.6	5.08	0.56
R357	NE	IdoA2(F) - COO^-	C6	40.4	5.11	0.55
R357	CZ	IdoA2(F) - 2 - OSO_3^-	S1	39.9	4.88	0.49
R357	CZ	IdoA2(F) - COO^-	C6	36.3	4.95	0.63
R357	NE	GlcNS,6S(E) - $NHSO_3^-$	S1	33.7	5.09	0.64
R357	NE	IdoA2(F) - 2 - OSO_3^-	S1	33.3	5.48	0.33
K356	NZ	GlcNS,6S(C) - 6 - OSO_3^-	S1	32.8	4.50	0.79
R466	CZ	IdoA2S(B) - COO^-	C6	26.9	4.33	0.36
R466	NE	IdoA2S(B) - COO^-	C6	25.0	5.53	0.19
R346	CZ	GlcNS,6S(A) - 6 - OSO_3^-	S1	24.4	4.64	0.47
R357	CZ	GlcNS,6S(E) - $NHSO_3^-$	S1	23.8	5.17	0.66
K356	NZ	GlcNS,6S(C) - $NHSO_3^-$	S1	20.8	4.45	0.89
R346	NE	GlcNS,6S(A) - $NHSO_3^-$	S1	20.0	4.99	0.77
R346	CZ	GlcNS,6S(A) - $NHSO_3^-$	S1	19.6	4.75	0.58
R346	NE	GlcNS,6S(A) - 6 - OSO_3^-	S1	19.5	4.92	0.83
K356	NZ	IdoA2S(B) - COO^-	C6	19.2	4.66	0.95
K356	NZ	GlcNS,6S(E) - $NHSO_3^-$	S1	15.4	4.13	0.73
R357	NE	GlcNS,6S(C) - 6 - OSO_3^-	S1	11.8	4.78	0.80

Table b

Protein Residue	Atom name	Ligand residue	Atom name	Percentage (%)	Distance (A°)	Dev. Std (A°)
R357	CZ	IdoA2(F) - COO^-	O3	65.8	4.01	0.63
R357	NE	IdoA2(F) - COO^-	O3	63.5	3.63	0.76
N394	ND2	IdoA2(F) -O^-	O3	50.9	3.68	0.41
Y396	OH	IdoA2(F) - COO^-	O3	43.7	3.11	0.44
R357	N	GlcNS,6S(C) - 6 - OSO_3^-	S1	30.8	3.91	0.27
N394	ND2	IdoA2(F) - 2 - OSO_3^-	S1	25.1	4.02	0.23
Y396	OH	GlcNS,6S(E) - 6 - OSO_3^-	S1	22.3	3.92	0.55
S359	OG	GlcA(D)- COO^-	C6	20.9	3.73	0.33

N354	ND2	IdoA2S(B) - COO^-	C6	16.3	3.78	0.28
R357	N	GlcNS,6S(E) - $NHSO_3^-$	S1	13.8	4.04	0.17
N394	ND2	GlcNS,6S(E) - 6 - OSO_3^-	S1	12.4	3.99	0.26
N360	ND2	GlcNS,6S(C) - $NHSO_3^-$	S1	11.8	4.14	0.21
S349	OG	GlcNS,6S(A) - $NHSO_3^-$	S1	11.7	3.65	0.22
S349	N	GlcNS,6S(A) - $NHSO_3^-$	S1	11.5	3.78	0.27
Y396	OH	IdoA2(F) - COO^-	C6	10.8	3.70	0.27

Table c

Protein Residue	Atom name	Ligand residue	Atom name	Percentage (%)	Distance (Å)	Dev. Std (Å)
R346	NE	GlcA(D)- COO^-	C6	67.7	4.81	0.867
R346	CZ	GlcA(D)- COO^-	C6	67.6	4.5	0.652
R346	NE	GlcNS,6S(C) - 6 - OSO_3^-	S1	50.6	4.55	0.849
R346	CZ	GlcNS,6S(C) - $NHSO_3^-$	S1	50.3	4.78	0.502
R346	CZ	GlcNS,6S(C) - 6 - OSO_3^-	S1	43.7	4.67	0.598
R346	NE	GlcNS,6S(C) - $NHSO_3^-$	S1	41.5	4.96	0.816
K356	NZ	IdoA2S(B) - COO^-	C6	39.6	4.32	0.791
K356	NE	GlcNS,6S(A) - $NHSO_3^-$	S1	29.9	4.1	0.671
K357	CZ	GlcNS,6S(A) - $NHSO_3^-$	S1	26.8	4.47	0.418
K356	NZ	GlcNS,6S(C) - 6 - OSO_3^-	S1	25.5	4.47	0.77
K444	NZ	IdoA2(F) - 2 - OSO_3^-	S1	21.9	4.21	0.701
R466	CZ	GlcNS,6S(E) - 6 - OSO_3^-	S1	20.2	4.51	0.469
K444	NZ	IdoA2(F) - COO^-	C6	18	3.94	0.772
R466	NE	GlcNS,6S(E) - 6 - OSO_3^-	S1	17.5	5.4	0.472
R355	NE	GlcNS,6S(E) - 6 - OSO_3^-	S1	16.1	5.27	0.431
K444	NZ	GlcNS,6S(E) - 6 - OSO_3^-	S1	14.6	4.14	0.594
R466	CZ	IdoA2(B) - 2 - OSO_3^-	S1	12.1	4.81	0.486
R466	CZ	GlcNS,6S(C) - $NHSO_3^-$	S1	10.6	4.32	0.223
R466	NE	GlcNS,6S(C) - $NHSO_3^-$	S1	10.2	5.57	0.137

Table d

Protein Residue	Atom name	Ligand residue	Atom name	Percentage (%)	Distance (Å)	Dev. Std (Å)
T345	N	GlcNS,6S(C) - 6 - OSO_3^-	S1	49.3	3.8	0.3
T345	OG1	GlcNS,6S(C) - 6 - OSO_3^-	S1	43.1	3.77	0.29
R346	N	GlcNS,6S(C) - 6 - OSO_3^-	S1	39.2	4.15	0.19
A344	N	GlcNS,6S(C) - 6 - OSO_3^-	S1	38.2	5.41	0.318
K444	NZ	IdoA2(F) - COO^-	O3	16.1	4.81	1
N354	ND2	GlcNS,6S(C) - $NHSO_3^-$	S1	15.5	3.94	0.22
N354	ND2	IdoA2S(B) - COO^-	C6	13.8	3.88	0.23

Table e

Protein Residue	Atom name	Ligand residue	Atom name	Percentage (%)	Distance (A°)	Dev. Std (A°)
R346	CZ	GlcA(D)- COO^-	C6	84.6	4.63	0.45
R346	NE	GlcA(D)- COO^-	C6	79.9	4.87	0.58
K356	NZ	GlcNS,6S(C) - 6 - OSO_3^-	S1	72.6	4.30	0.66
R346	NE	GlcNS,6S(E) - $NHSO_3^-$	S1	57.0	5.33	0.54
R357	CZ	IdoA2(B) - 2 - OSO_3^-	S1	57.0	4.66	0.28
R357	NE	IdoA2(B) - 2 - OSO_3^-	S1	56.9	4.84	0.46
R357	CZ	GlcNS,6S(C) - 6 - OSO_3^-	S1	55.8	4.55	0.33
K356	NZ	GlcA(D)- COO^-	C6	54.6	3.74	0.46
R357	NE	GlcNS,6S(A) - 6 - OSO_3^-	S1	41.7	5.65	0.28
R466	CZ	GlcNS,6S(A) - 6 - OSO_3^-	S1	37.5	4.31	0.25
R346	NE	IdoA2(F) - COO^-	C6	37.1	5.05	0.64
R346	CZ	IdoA2(F) - COO^-	S1	36.3	5.11	0.64
R357	NE	GlcNS,6S(A) - $NHSO_3^-$	S1	35.8	4.08	0.63
R466	NE	GlcNS,6S(A) - 6 - OSO_3^-	S1	35.4	5.53	0.27
K356	NZ	GlcNS,6S(E) - $NHSO_3^-$	S1	34.5	4.74	0.69
K356	NZ	IdoA2S(B) - COO^-	C6	33.1	4.09	0.68
R357	CZ	GlcNS,6S(A) - $NHSO_3^-$	S1	32.7	4.52	0.41
R355	NE	GlcNS,6S(A) - 6 - OSO_3^-	S1	31.1	5.22	0.38
R346	NE	GlcNS,6S(C) - 6 - OSO_3^-	S1	30.2	4.10	0.66
R346	CZ	GlcNS,6S(C) - 6 - OSO_3^-	S1	29.2	4.47	0.45
R346	CZ	IdoA2(F) - COO^-	C6	27.6	5.13	0.63
R355	CZ	GlcNS,6S(A) - 6 - OSO_3^-	S1	19.6	5.43	0.44
R346	CZ	GlcNS,6S(E) - 6 - OSO_3^-	S1	18.5	4.89	0.56
R466	CZ	IdoA2(B) - 2 - OSO_3^-	S1	18.4	4.98	0.49
R466	NE	IdoA2(B) - 2 - OSO_3^-	S1	14.0	5.21	0.41
R346	NE	GlcNS,6S(E) - 6 - OSO_3^-	S1	10.2	5.38	0.58

Table f

Protein Residue	Atom name	Ligand residue	Atom name	Percentage (%)	Distance (A°)	Dev. Std (A°)
R346	CZ	IdoA2(F) - COO^-	O3	58.1	4.24	0.74
R346	NE	IdoA2(F) - COO^-	O3	55.0	4.27	0.76
R357	N	GlcNS,6S(C) - $NHSO_3^-$	S1	54.7	4.02	0.13
T345	N	GlcNS,6S(E) - $NHSO_3^-$	S1	48.0	3.81	0.27
R346	N	GlcNS,6S(E) - $NHSO_3^-$	S1	42.0	4.09	0.20
T345	OG1	GlcNS,6S(E) - $NHSO_3^-$	S1	32.9	3.84	0.33
N354	ND2	IdoA2S(B) - COO^-	C6	27.2	3.80	0.16

T345	OG1	IdoA2(F) - COO^-	C6	24.4	3.67	0.43
N360	ND2	GlcNS,6S(A) - 6 - OSO_3^-	S1	18.5	3.99	0.30

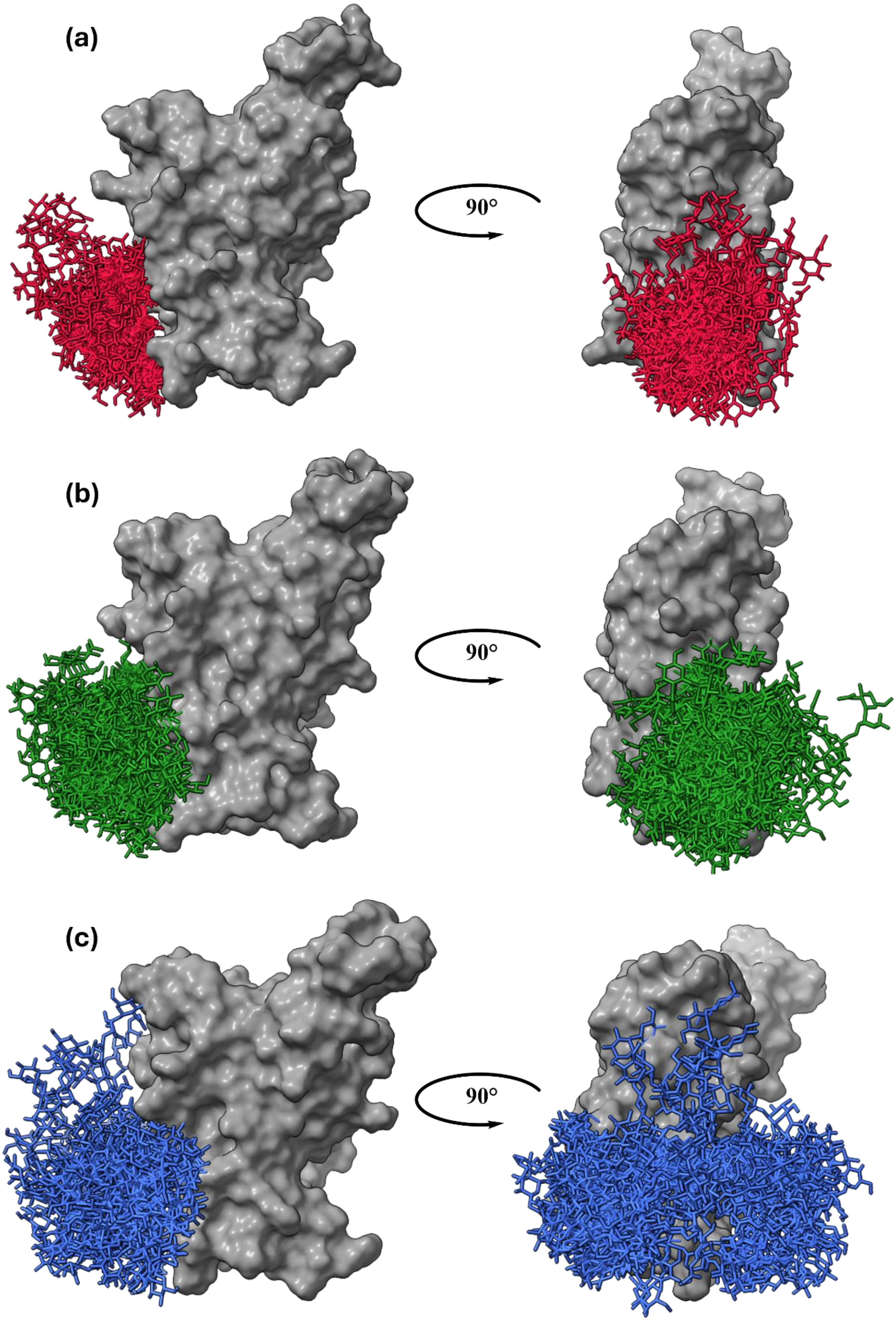

Figure S11. Graphical representation of the conformation populated by the FA2G2 glycan at N343 in Omi-S1-RBD-hexa-I (a), Omi-S1-RBD-hexa-II (b), and WT-S1-RBD-hexa (c). The FA2G2 geometries were sampled at an interval of 10 ns for a total of 60 snapshots. The protein is depicted as a grey surface, while the FA2G2 glycan is in a stick (red, green and blue for Omi-S1-RBD-hexa-I, Omi-S1-RBD-hexa-II, and WT-S1-RBD-hexa, respectively).

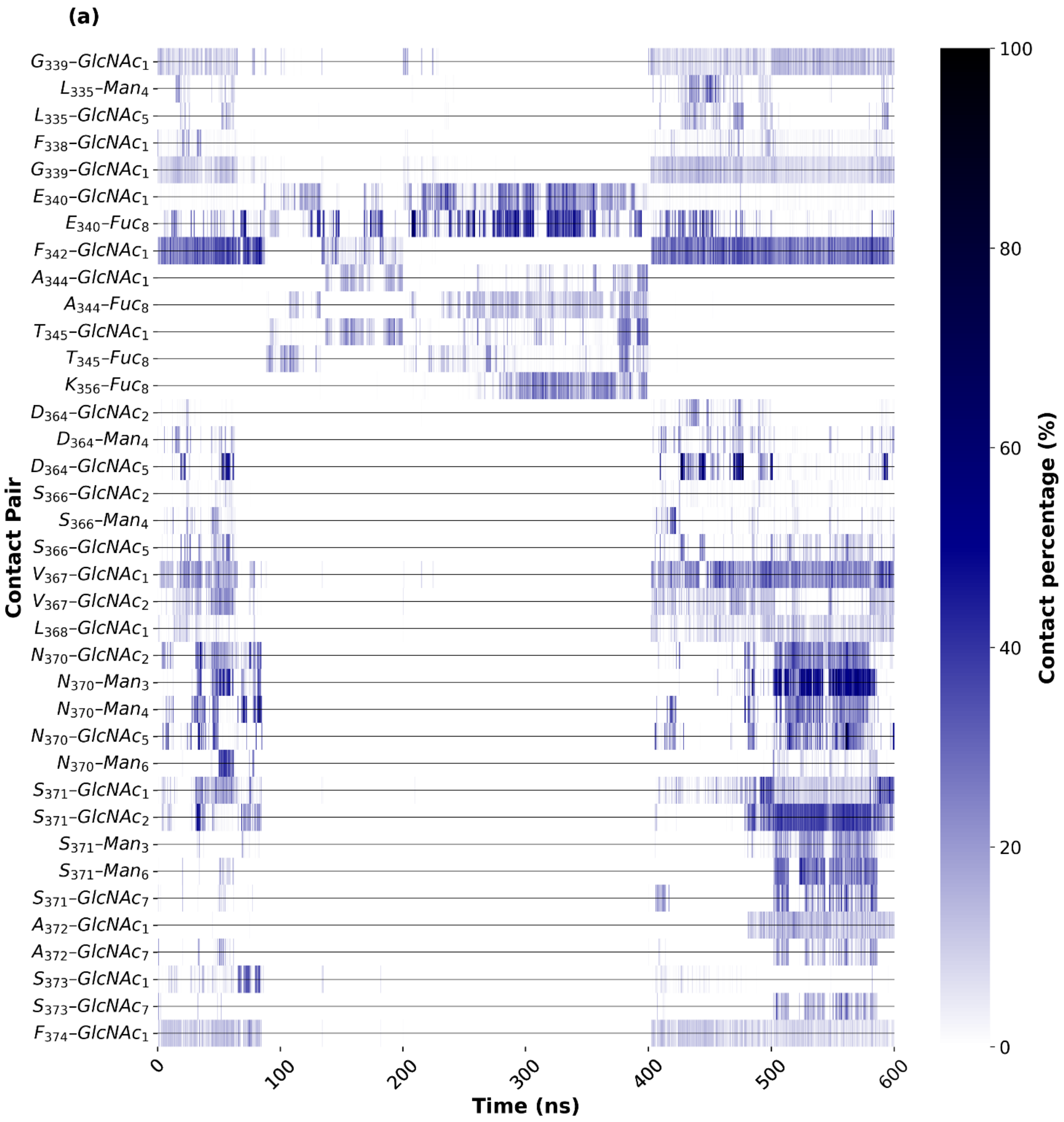
(a)
G_{339}–$GlcNAc_1$
L_{335}–Man_4
L_{335}–$GlcNAc_5$
F_{338}–$GlcNAc_1$
G_{339}–$GlcNAc_1$
E_{340}–$GlcNAc_1$
E_{340}–Fuc_8
F_{342}–$GlcNAc_1$
A_{344}–$GlcNAc_1$
A_{344}–Fuc_8
T_{345}–$GlcNAc_1$
T_{345}–Fuc_8
K_{356}–Fuc_8
D_{364}–$GlcNAc_2$
D_{364}–Man_4
D_{364}–$GlcNAc_5$
S_{366}–$GlcNAc_2$
S_{366}–Man_4
S_{366}–$GlcNAc_5$
V_{367}–$GlcNAc_1$
V_{367}–$GlcNAc_2$
L_{368}–$GlcNAc_1$
N_{370}–$GlcNAc_2$
N_{370}–Man_3
N_{370}–Man_4
N_{370}–$GlcNAc_5$
N_{370}–Man_6
S_{371}–$GlcNAc_1$
S_{371}–$GlcNAc_2$
S_{371}–Man_3
S_{371}–Man_6
S_{371}–$GlcNAc_7$
A_{372}–$GlcNAc_1$
A_{372}–$GlcNAc_7$
S_{373}–$GlcNAc_1$
S_{373}–$GlcNAc_7$
F_{374}–$GlcNAc_1$
Contact Pair
0
100
200
300
400
500
600
Time (ns)
100
80
60
40
20
0
Contact percentage (%)

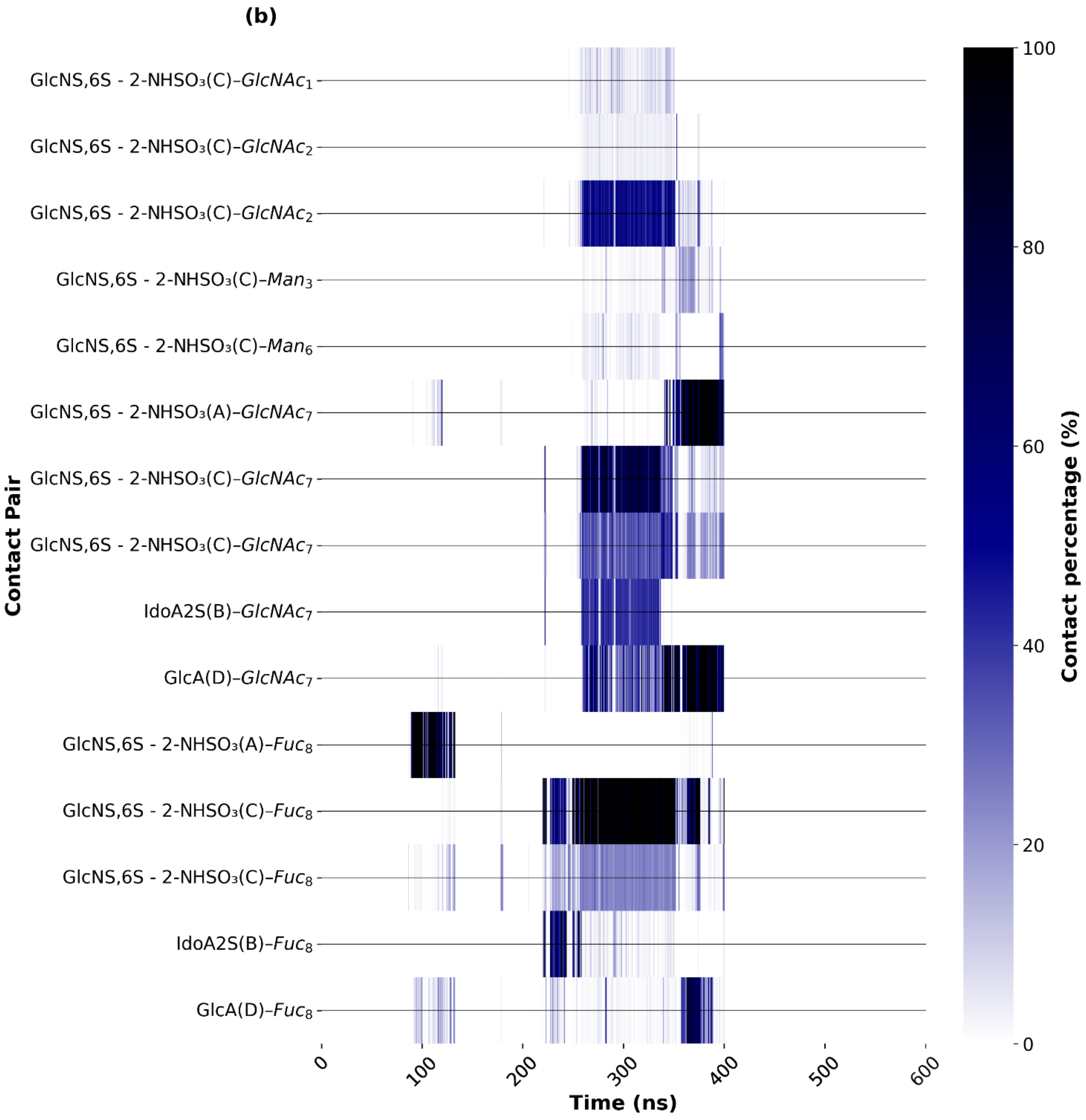

(b)
GlcNS,6S - 2-NHSO₃(C)–GlcNAc₁
GlcNS,6S - 2-NHSO₃(C)–GlcNAc₂
GlcNS,6S - 2-NHSO₃(C)–GlcNAc₂
GlcNS,6S - 2-NHSO₃(C)–Man₃
GlcNS,6S - 2-NHSO₃(C)–Man₆
GlcNS,6S - 2-NHSO₃(A)–GlcNAc₇
GlcNS,6S - 2-NHSO₃(C)–GlcNAc₇
GlcNS,6S - 2-NHSO₃(C)–GlcNAc₇
IdoA2S(B)–GlcNAc₇
GlcA(D)–GlcNAc₇
GlcNS,6S - 2-NHSO₃(A)–Fuc₈
GlcNS,6S - 2-NHSO₃(C)–Fuc₈
GlcNS,6S - 2-NHSO₃(C)–Fuc₈
IdoA2S(B)–Fuc₈
GlcA(D)–Fuc₈
Contact Pair
0
100
200
300
400
500
600
Time (ns)
0
20
40
60
80
100
Contact percentage (%)

(c)

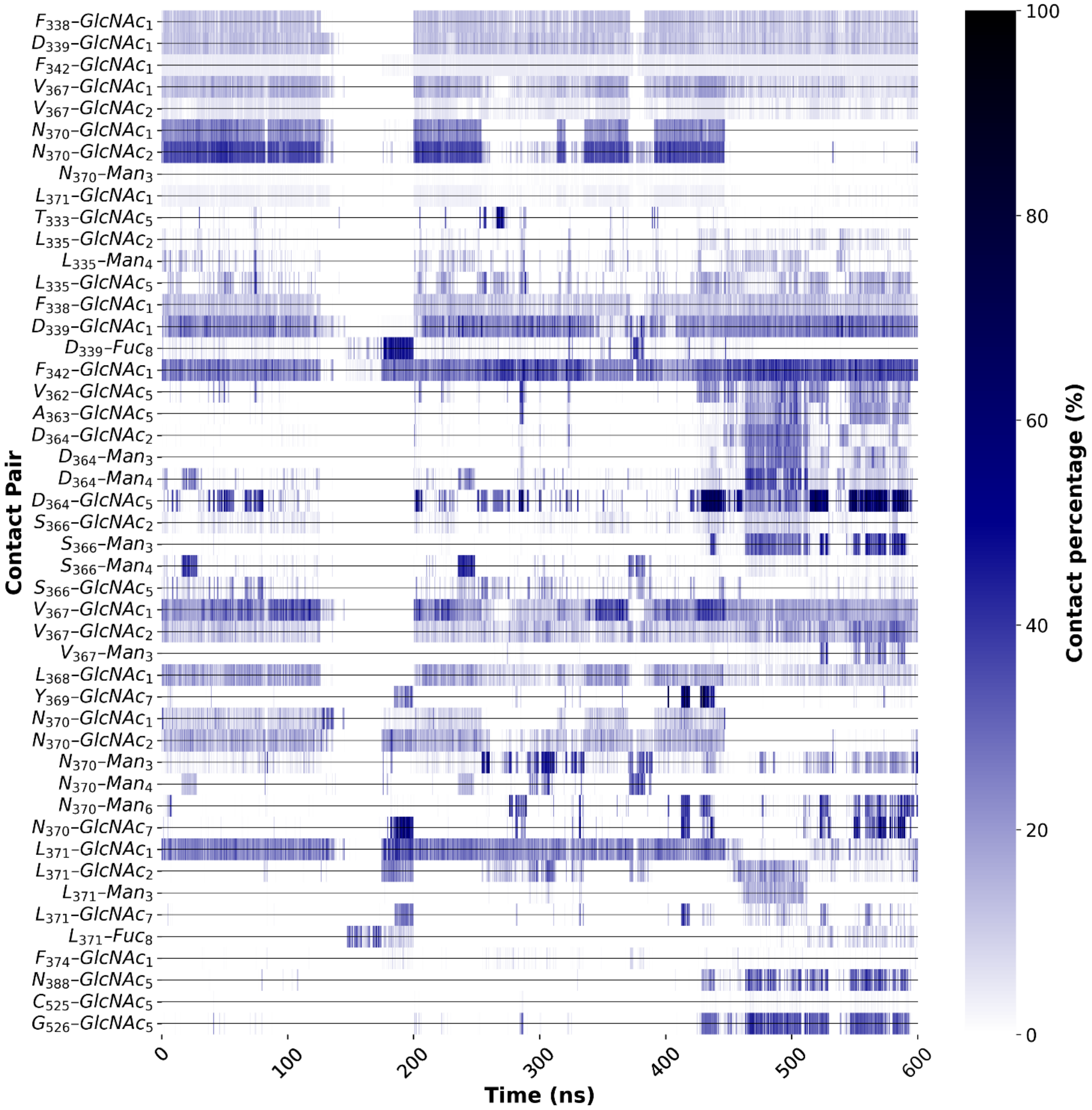
F338–GlcNAc1
D339–GlcNAc1
F342–GlcNAc1
V367–GlcNAc1
V367–GlcNAc2
N370–GlcNAc1
N370–GlcNAc2
N370–Man3
L371–GlcNAc1
T333–GlcNAc5
L335–GlcNAc2
L335–Man4
L335–GlcNAc5
F338–GlcNAc1
D339–GlcNAc1
D339–Fuc8
F342–GlcNAc1
V362–GlcNAc5
A363–GlcNAc5
D364–GlcNAc2
D364–Man3
D364–Man4
D364–GlcNAc5
S366–GlcNAc2
S366–Man3
S366–Man4
S366–GlcNAc5
V367–GlcNAc1
V367–GlcNAc2
V367–Man3
L368–GlcNAc1
Y369–GlcNAc7
N370–GlcNAc1
N370–GlcNAc2
N370–Man3
N370–Man4
N370–Man6
N370–GlcNAc7
L371–GlcNAc1
L371–GlcNAc2
L371–Man3
L371–GlcNAc7
L371–Fuc8
F374–GlcNAc1
N388–GlcNAc5
C525–GlcNAc5
G526–GlcNAc5
Contact Pair
0
100
200
300
400
500
600
Time (ns)
100
80
60
40
20
0
Contact percentage (%)

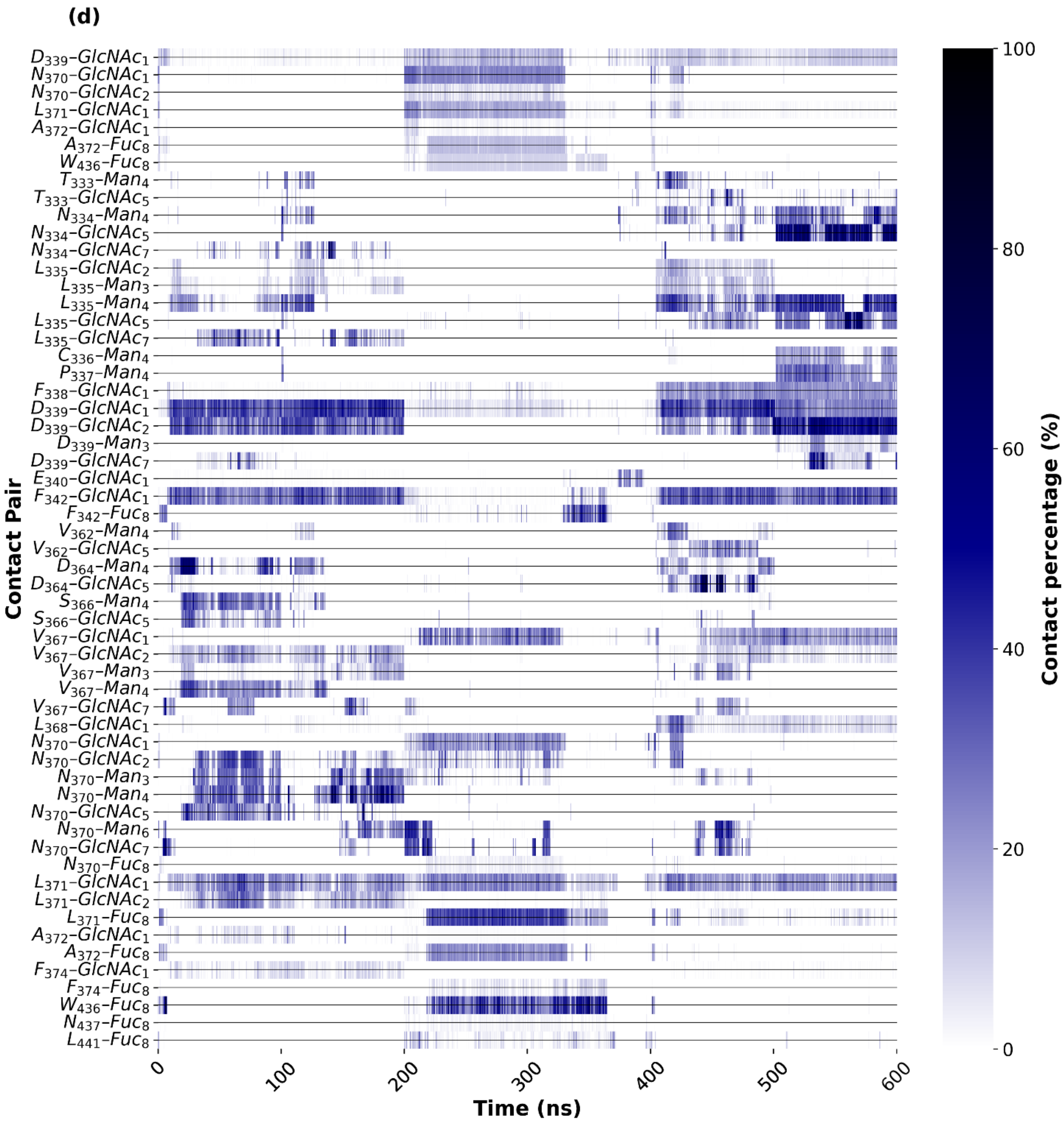

Figure S12. Heatmap of contacts observed between hexa and FA2G2 and between FA2G2 and S1-RBD in WT-S1-RBD-hexa (**a**, **b**) and Omi-S1-RBD-hexa-I (**c**) and RBD-Omi-hexa-II (d) meta-trajectories. For clarity, any contact with a global percentage (calculated as the number of contacts formed over 600 ns) of less than 10% is omitted from the heatmap.

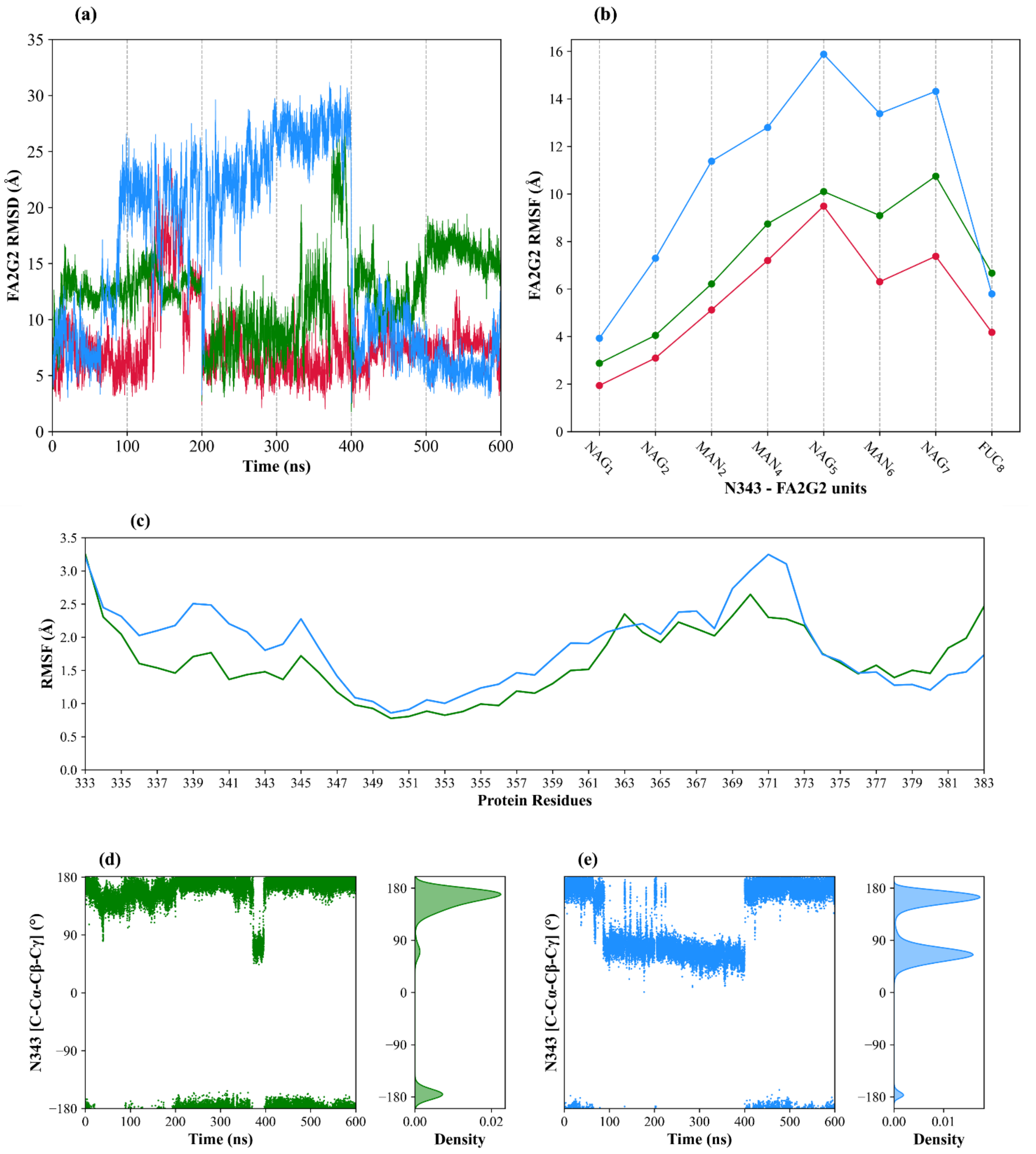

Figure S13. Conformational analysis of FA2G2 in Omi-S1-RBD-hexa-I (red), Omi-S1-RBD-hexa-II (green) and WT-S1-RBD-hexa (blue) concatenated simulations. (a) RMSD analysis of octasaccharide backbone, calculated over time. (b) RMSF plot for each monosaccharide unit of FA2G2 calculated over a combined 600 ns. (c) RMSF analysis of residue 333-383 of RBD protein, calculated relative to frame 1 (t=0) of the combined trajectories. (d) Scatter plot of χ_1 torsion of the N343 side chain, defined as $C - C_\alpha - C_\beta - C_\gamma$ for Omi-S1-RBD-Hexa-II. (e) Scatter plot of χ_1 torsion of the N343 side chain

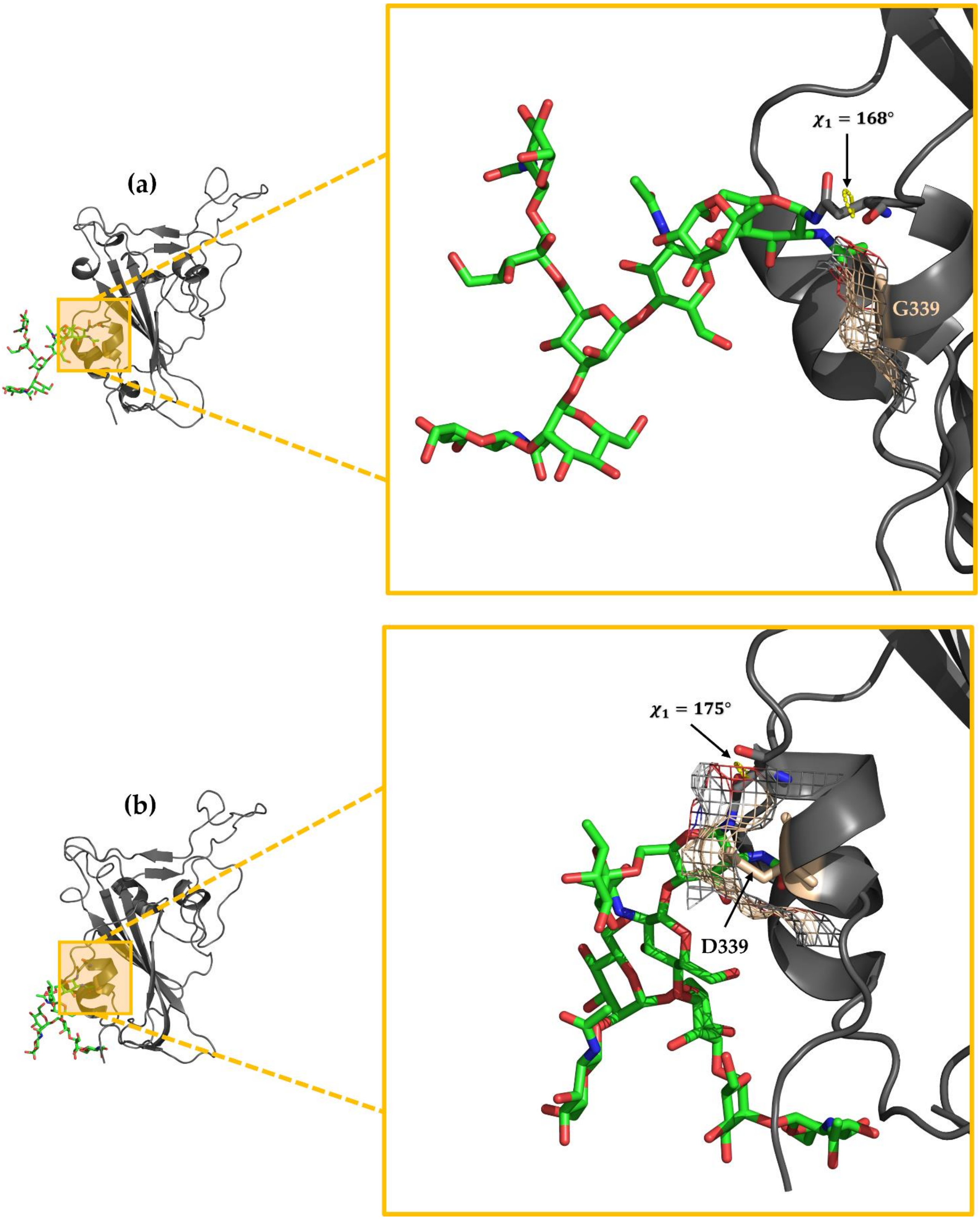

Figure S14. The G339D mutation in Omicron S1-RBD reduces FA2G2 glycan flexibility. The S1-RBD is shown as a grey cartoon, with FA2G2 in stick representation (grey carbons, red oxygens, blue nitrogens). Residue 339 is highlighted in light pink (Gly in wildtype, Asp in Omicron). In the wild type (**panel a**), G339, a small and apolar residue allows FA2G2 to explore multiple orientations, transitioning from *trans* ($\chi_1 \approx 160°$) to gauche+ ($\chi_1 \approx 60°$) state. In the Omicron variant (**panel b**), the bulkier, negatively charged D339 creates steric and electrostatic clashes with the GlcNAc[1] and Fuc[8] residues, restricting glycan motion to the *trans* state.

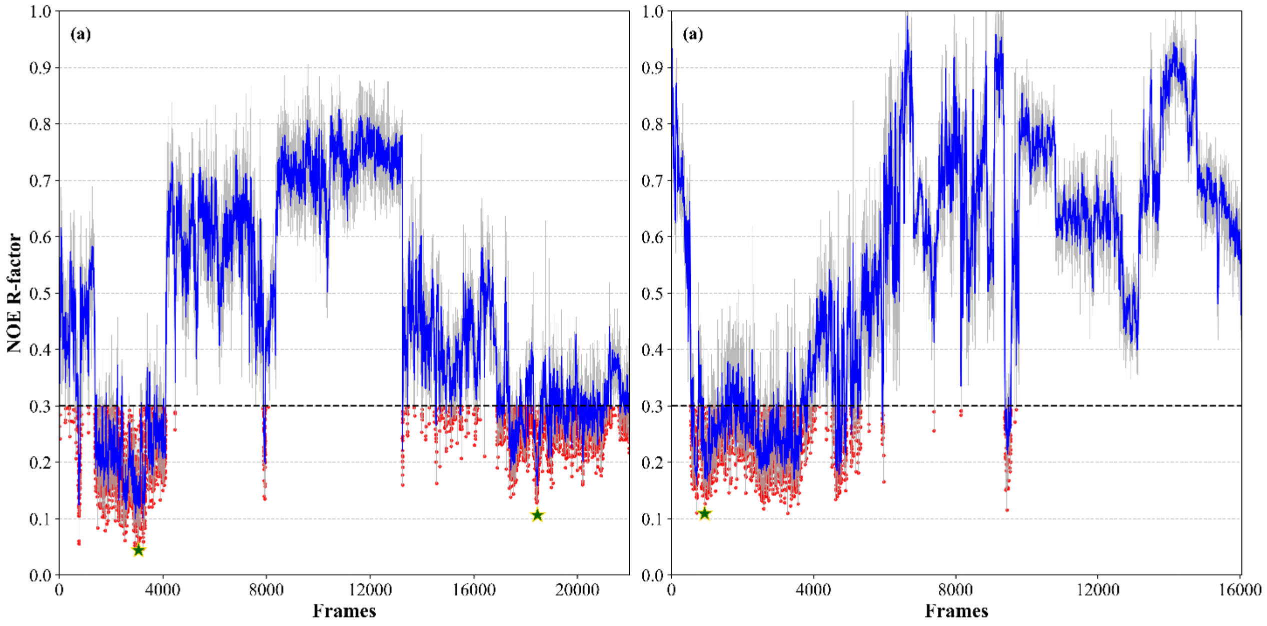

Figure S15. Evolution of the R-NOE factor for RBD-Omi-hexa-I (a) and RBD-Omi-hexa-II (b) over the metatrajectory. The grey line represents the R-NOE factor trend, while the blue line shows the rolling average (window size = 10). Structures with R-NOE values below 0.3 (indicating strong agreement between experimental and theoretical binding epitopes) are marked with red dots. The frame with the lowest R-NOE value is highlighted with a green star.

Table S5. Cluster analysis result of Omi-RBD-hexa-I (table a) and Omi-RBD-hexa-II (table b) and WT-RBD-hexa (table c), reporting the members and the relative percentage (perc. %) for the first five families determined. The calculation was performed on frames that exhibit an R-NOE factor lower than 0.3 for in Omi-RBD metatrajectories, while for the WT-RBD system the analysis was performed on the "strong binding" metatrajectory. Clusters are generated using the hierarchical agglomerative approach, setting a distance of 4.5Å between the clusters. The distance is calculated on hexa heavy atoms, considering the distance as the average distance between members of two clusters

(a)		
**Cluster**	**Frames**	**Perc. (%)**
**1**	2366	43.3
**2**	2129	39.0
**3**	439	8.0
**4**	172	3.2
**5**	146	2.7

(b)		
**Cluster**	**Frames**	**Perc. (%)**
**1**	2130	73.0
**2**	290	9.9
**3**	137	4.7
**4**	129	4.4
**5**	61	2.1

(c)		
**Cluster**	**Frames**	**Perc. (%)**
**1**	5692	48.2
**2**	2398	20.3
**3**	921	7.8
**4**	574	4.9
**5**	456	3.9

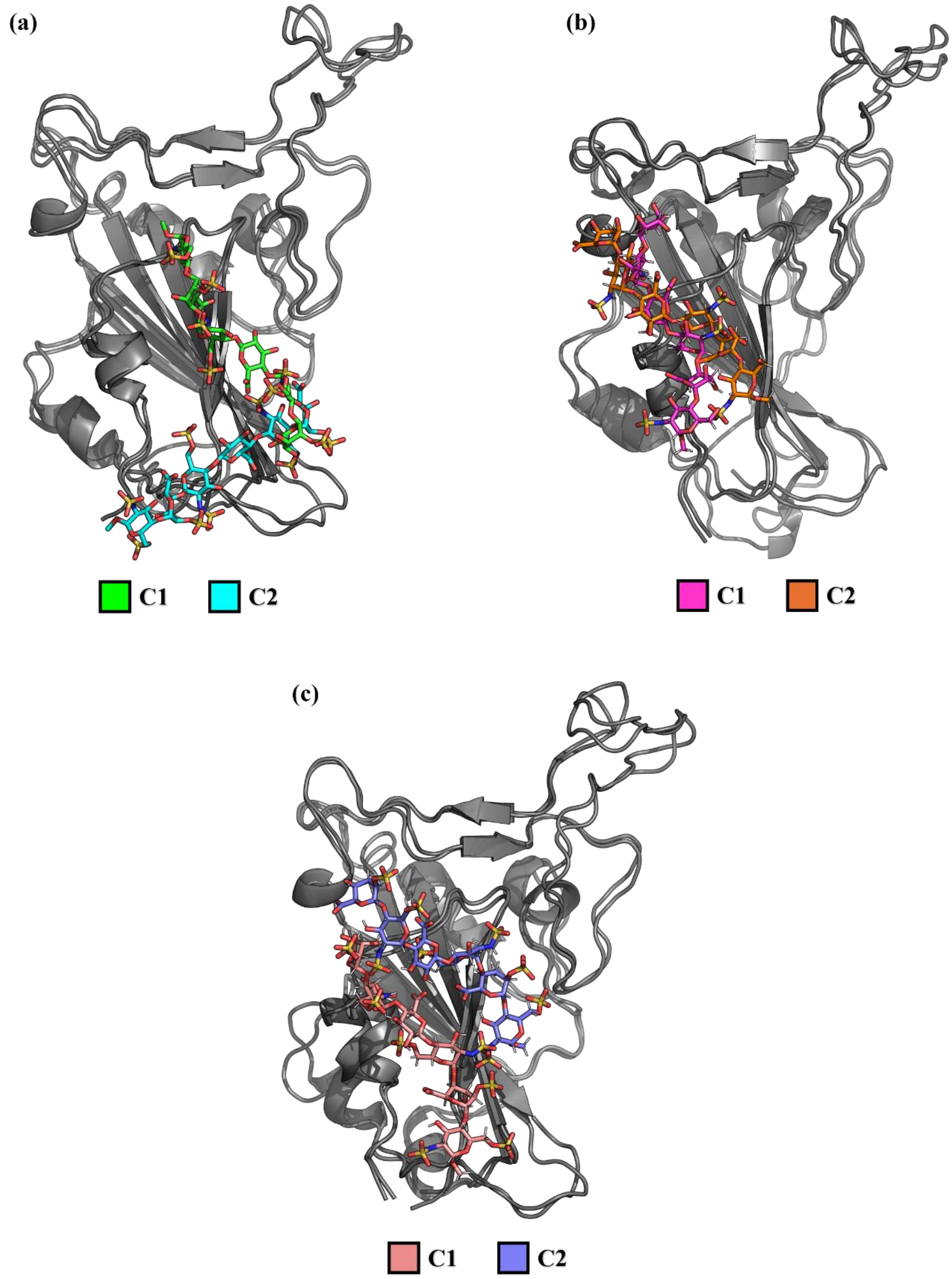

Figure S16. Representative clusters of Omi-RBD-hexa-I (a) and Omi-RBD-hexa-II (b) and WT-RBD-hexa (c) are depicted as stick using different colours as reported in the legend
